# Isatin Bis-Indole and Bis-Imidazothiazole Hybrids: Synthesis and Antimicrobial Activity

**DOI:** 10.3390/molecules27185781

**Published:** 2022-09-07

**Authors:** Francesca Bonvicini, Alessandra Locatelli, Rita Morigi, Alberto Leoni, Giovanna Angela Gentilomi

**Affiliations:** 1 Department of Pharmacy and Biotechnology, Alma Mater Studiorum-University of Bologna, Via Massarenti 9, 40138 Bologna, Italy; 2 Department of Pharmacy and Biotechnology, Alma Mater Studiorum-University of Bologna, Via Belmeloro 6, 40126 Bologna, Italy; 3 Division of Microbiology, IRCCS Azienda Ospedaliero-Universitaria di Bologna, Via Massarenti 9, 40138 Bologna, Italy

**Keywords:** isatin, indole, imidazo[2,1-*b*]thiazole, antimicrobial agents, cytotoxicity

## Abstract

Isatin and its derivatives are important heterocycles found in nature and present in numerous bioactive compounds which possess various biological activities. Moreover, it is an essential building block in organic synthesis. The discovery of novel compounds active against human pathogenic bacteria and fungi is an urgent need, and the isatin may represent the suitable scaffold in the design of biologically relevant antimicrobials. A small library of 18 isatin hybrids was synthetized and evaluated for their antimicrobial potential on three reference strains: *S. aureus*, *E. coli*, both important human pathogens infamous for causing community- and hospital-acquired severe systemic infections; and *C. albicans*, responsible for devastating invasive infections, mainly in immunocompromised individuals. The study highlighted two lead compounds, **6k** and **6m,** endowed with inhibitory activity against *S. aureus* at very low concentrations (39.12 and 24.83 µg/mL, respectively).

## 1. Introduction

The exploration of privileged heterocyclic frameworks is one of the most significant areas in drug discovery, and isatin is an essential building block in organic synthesis.

Isatin (indoline-2,3-dione) and its derivatives are important heterocycles found in nature and present in numerous bioactive compounds, which can act as anticancer, antitubercular, anti-HIV, antimalarial, and antimicrobial agents [1]. The most attractive application of isatin in organic synthesis is undoubtedly in the highly reactive C-3 carbonyl group that readily undergoes condensation reaction, but the N-1 and C-5 positions are also domains of chemical variations.

In recent years, various isatin derivatives have been screened for their antimicrobial activities, and some of them demonstrated promising in vitro and in vivo activity [2].

Even the imidazo[2,1-*b*]thiazole ring is an attractive fused heterocyclic system containing an imidazole ring fused to a thiazole ring by means of a bridgehead nitrogen atom that has been extensively studied because of its different biological activities as immunomodulatory, antifungal, anthelmintic, antimicrobial, and antitumour agents reported in literature in recent years [3,4,5,6,7,8,9,10,11,12].

Molecular hybridization involves a combination of pharmacophore moieties/bioactive substances to produce a novel compound with better efficacy than potent substrates [13,14,15].

With this in mind and on the basis of our experience on imidazo[2,1-*b*]thiazoles and indolinones chemistry, we have designed a new series of isatin bis-indole and isatin bis-imidazothiazole hybrids; specifically, in the isatin moiety almost all positions have been modified, while only in some cases the indoles have been decorated with a methoxy group in position 5, and the imidazothiazole was maintained unaltered.

The herein synthetized isatin derivatives were screened in vitro for their antibacterial and antifungal activity. In particular, the potential of the compounds was investigated against *Staphylococcus aureus* and *Escherichia coli* as representative Gram-positive and Gram-negative bacteria, and *Candida albicans* as a yeast model.

*S. aureus* and *E. coli* are important human pathogens of concern, infamous for causing community- and hospital-acquired infections with high mortality rates in the case of severe systemic infections. Indeed, they are common causes of bacterial infections, but they can also enter the bloodstream and reach different organs and tissues of the human body, thereby causing metastatic infections [16]. The treatment of such infections, unfortunately, has become even more difficult due to the emergence of multiple drug resistance. As for *S. aureus*, this is best exemplified by the methicillin-resistant *S. aureus* (MRSA) strains. Regarding *E. coli*, resistance to critically important antimicrobials, such as third/fourth/fifth-generation cephalosporins and quinolones, and multidrug resistance (MDR) are recognized globally. Recently, the relevance of fungi as human pathogens has been widely recognized because many species, including *C. albicans*, are responsible for devastating invasive infections, mainly in immunocompromised individuals, such as patients subjected to organ transplantation and those affected by cancer or AIDS. Antifungal therapy failure as well is gradually increasing worldwide because of the diffuse resistance to routinely used antifungal drugs, namely polyenes, azoles, and echinocandins [17].

Combined, the discovery of novel compounds active against human pathogenic bacteria and fungi is an urgent need, and the isatin may represent the suitable scaffold in the design of biologically relevant antimicrobials.

## 2. Results and Discussion

### 2.1. Chemistry

**Scheme 1:**
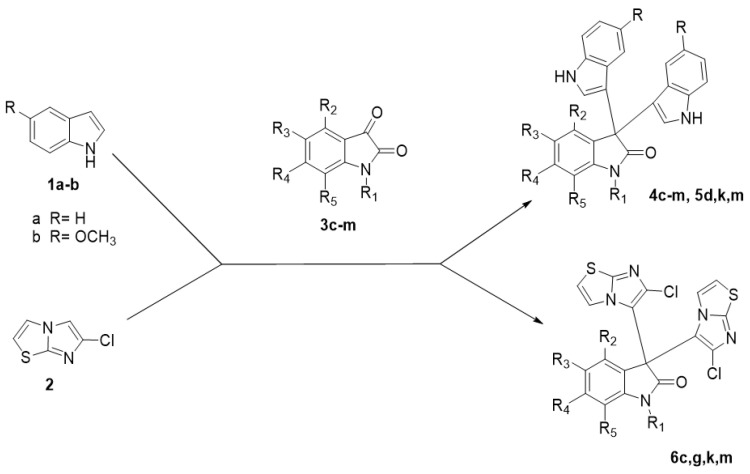
Synthesis of new derivatives, **4c-m**; **5d**,**k**,**m**; **6c**,**g**,**k**,**m**.

The synthetic route to the designed compounds **4**–**6** is reported in Scheme 1.

For R–R_5_ see Table 1.

The new compounds were obtained by reacting isatins with indoles or imidazo[2,1-*b*]thiazoles in isopropanol in the presence of molecular iodine (Scheme 1). Indoles **1a-b** are commercially available. The 6-Chloroimidazo[2,1-*b*]thiazole **2** has been prepared as described in the literature [20]. The isatins, **3c-m**, were either commercially available or prepared via the Sandmeyer reaction of aniline derivatives with chloral hydrate and hydroxylamine hydrochloride followed by cyclization of the resulting hydroxyiminoacetanilide intermediates by heating in concentrated sulfuric acid [21,22,23,24,25,26,27,28,29].

The structures of the final compounds were confirmed by means of ^1^H-NMR, ^13^C-NMR and HRMS spectra.

The analysis of the ^1^H-NMR spectra allowed us to make some considerations. The derivatives with the isatin core bearing two indole groups have a plane of symmetry so that the protons of the two indoles are in the same chemical environment. In fact, for each proton of the two indoles, the spectra show only one signal integrating for two protons, thus confirming that the two indoles are equivalent.

Moreover, in most of these derivatives a coupling between the indole NH and the proton in position 2 (ind2) is observed, which gives rise to two doublets having the same J. In some cases, the NH signal is a broad singlet, but the coupling is confirmed in the D_2_O exchange spectra: the indole NH protons exchange and the ind2 doublet becomes a singlet.

Conversely, the compounds **6** with the isatin core bearing two imidazothiazoles have no plane of symmetry; thus, the imidazothiazoles are in different chemical environments. Indeed, each imidazothiazole proton gives its own signal, and in compounds **6g** and **6m**, the benzylic methylene protons form two coupling doublets instead of a singlet because they are diastereotopic.

A bibliographic survey performed using Reaxys database (http://www.reaxys.com (accessed on 24 July 2022)) showed that the synthesis of the compounds, **4c**, **4j-k**, and **5k**, were already reported [18,19].

### 2.2. Biological Evaluation

The multi-substituted isatin derivatives were assayed in vitro against *Staphylococcus aureus* ATCC 25923, *Escherichia coli* ATCC 25922, and *Candida albicans* ATCC 10231. These strains were selected as representative Gram-positive, Gram-negative, and fungal species responsible for a broad spectrum of human infections. All compounds were tested at 100 μM against the three microbial species and on epithelial cells (Vero, ATCC CCL-81) to measure their ability to affect mammalian cell metabolism.

At first glance at the data reported in Table 2 and Figure S1, some general remarks can be drawn. As for the antibacterial activity, the tested compounds interfered with *S. aureus* and *E. coli* growths to varying degrees; indeed, several derivatives reduced *S. aureus* proliferation, and compounds **4c**, **6k** and **6m** proved to be highly active against this Gram*-positive* strain. Conversely, none of them displayed inhibitory properties toward *E. coli*. There are many examples of compounds, including clinically used antimicrobial drugs, which are antibacterial against Gram-positive organisms but which are ineffective against Gram-negative ones [30,31,32,33]. It is plausible that the differences in cell envelope structure would present differential barriers to penetration that would be reflected in divergent physicochemical property patterns for molecules effective against the two Gram species.

As for the antifungal activity, only **4h** significantly reduced the growth of *C. albicans* when tested at 100 µM (48.93 µg/mL), thus suggesting a generally high resistance of the yeast to the isatin derivatives. Indeed, literature reports scarce data concerning the antifungal properties of the isatin-based compounds [1], and the effectiveness of some small series of derivatives has been mainly demonstrated against filamentous fungi in the range of concentration 20–100 µg/mL [34].

In the frame of a comprehensive analysis on the antimicrobial potential of the isatin derivatives, all compounds were also evaluated for their cytotoxicity on the Vero cell line. These non-malignant cells are internationally recommended as a standard to study cytotoxicity; they have well-defined culturing characteristics in all experimental settings, thus suitable to define the overall safety of the isatin analogues [ISO 10993-5; Biological Evaluation of Medical Devices—Part 5: Tests for In Vitro Cytotoxicity. International Organization for Standardization: London, UK, 2009]. Generally, in vitro assessment of the toxicological profile of pure compounds as well as naturally inspired products must be part of the pipeline in a drug discovery perspective to discriminate a selective antimicrobial effect from a generic cytotoxicity on cells [35,36]. Specifically, as for isatin derivatives, these investigations are crucial as the anti-cancer activity of the isatin “building block” has been widely demonstrated [1,34]. As a matter of fact, in our experimental conditions, eight compounds significantly interfered with Vero metabolism, confirming the overall cytotoxicity of these compounds.

Comparing the antimicrobial activity of **4c**, **6k**, and **6m** with their cytotoxicity results, a selective inhibitory property against the Gram-positive strain was only confirmed for compounds **6k** and **6m**. Indeed, derivative **4c** strongly reduced *S. aureus* growth as well as Vero cell metabolism, thus excluding its specific antibacterial activity.

Overall, it seems that when the indolinone nucleus is linked to two indoles, the derivatives affect Vero cells proliferation, as in case of compounds **4c**, **4e**, **4f**, **4h**, and **4j-l**. On the other hand, the substitution of the two indole systems with the imidazothiazole nucleus, leading to compounds **6**, determined a lower cytotoxicity against Vero cells and at the same time, when the indolinone is properly substituted, a good efficacy versus *S. aureus* (compounds **6k** and **6m**).

**Figure 1:**
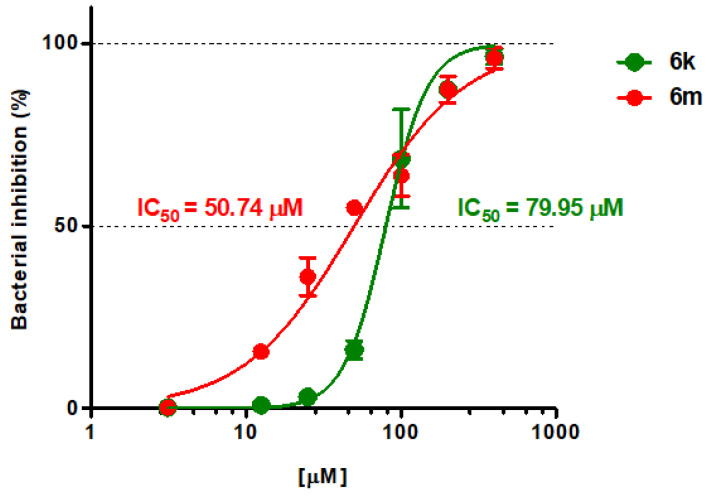
Dose−response curves of derivatives **6k** and **6m** against *S. aureus*. Symbols represent mean values with standard deviations, and lines define the curves obtained from nonlinear regression analysis. Percentage values are relative to the positive control.

Therefore, derivatives **6k** and **6m**, inhibiting *S. aureus* growth at a non-toxic concentration, were subjected to dose–response experiments to determine their IC_50_ values (Figure 1). The percentage values of the bacterial inhibition defined complete sigmoidal curves ranging from 0 to 100% response, thus indicating the efficacy of the derivatives to completely inhibit bacterial growth at the highest tested concentration (400 µM). Compound **6k** showed an IC_50_ of 79.95 µM (39.12 µg/mL) (95% confidence interval 73.15–87.38 µM), and compound **6m** showed an IC_50_ of 50.74 µM (24.83 µg/mL) (95% confidence interval 41.80–61.59 µM). By way of conclusion, these bis-imidazothiazole isatin hybrids demonstrated excellent antibacterial activity, higher than some isatin-azole hybrids [37] and isatin-coumarin hybrids [38], and close to certain isatin-carbohydrazides previously described [2].

Having demonstrated the antibacterial activity of **6k** and **6m** against the reference strain of *S. aureus*, 10 clinical isolates presenting different antibiotic susceptibilities and including both methicillin-sensitive (MSSA) and methicillin-resistant strains (MRSA) were tested (Table S1). Isatin derivatives proved to be effective toward MSSA as well as MRSA strains; it is worth noting that IC_50_ values obtained for the clinical isolates were close to those of the reference bacterial strain and ranging from 73.01 µM to 85.31 µM and from 45.02 µM to 59.38 µM for **6k** and **6m**, respectively.

## 3. Materials and Methods

### 3.1. Chemistry

The melting points are uncorrected. Elemental analyses were within ±0.4% of the theoretical values. Bakerflex plates (silica gel IB2-F) were used for TLC: the eluent was petroleum ether/acetone in various proportions. ^1^H-NMR and ^13^C-NMR spectra were recorded in (CD_3_)_2_SO on a Varian MR 400 MHz (ATB PFG probe); the chemical shift (referenced to solvent signal) is expressed in *δ* (ppm) and J in Hz; abbreviations: bn = benzyl, ind = indole; is = isatine; th = thiazole. High-resolution mass spectrometry (HRMS) data were analyzed by flow injection, utilizing electrospray ionization (ESI) on a Waters Xevo G2-XS QTOF (Milford, MA, USA) instrument in the positive mode. Compounds were named relying on the naming algorithm developed by CambridgeSoft Corporation (Perkin Elmer, Milan, Italy) and used in Chem-BioDraw Ultra 14.0 (Perkin Elmer, Milan, Italy). ^1^H-NMR, ^13^C-NMR, and HRMS spectra are reported as Supplementary Materials. All solvents and reagents, unless otherwise stated, were supplied by Aldrich Chemical Co. Ltd. (Milan, Italy) and were used without further purification.

Indoles **1a-b** are commercially available; 6-chloroimidazo[2,1-*b*]thiazole **2** has been prepared as described in the literature [20]. The isatins **3c-m** were either commercially available or prepared as described in the literature [21,22,23,24,25,26,27,28,29].

Compounds were not recognized by SwissADME software (Lousanne, Switzerland, 2022) [http://www.swissadme.ch/index.php] (accessed on 25 July 2022) as PAINS (Pan Assay INterference compoundS), i.e., molecules containing substructures showing potent response in assays irrespective of the target. This evidence allows to exclude that the studied derivatives interact nonspecifically and yield false positive biological output.

#### Synthesis of New Compounds, **4d-i**, **4l-m**, **5d**, **5m**, **6c**, **6g**, **6k**, and **6m**

Isatin (1.72 mmol) was dissolved in 20 mL of isopropanol taken in a reaction vessel, and iodine (5 mmol) was added to it. The appropriate indole or imidazo[2,1-*b*]thiazole (3.46 mmol) was added to the mixture, and the reaction was continued with constant stirring for 15 min–2 h (according to a TLC test). After completion of reaction, the mixture was concentrated under reduced pressure in a rotary evaporator. It was then extracted with chloroform, and the extract was washed with saturated sodium thiosulphate solution to decompose the remaining iodine. From the aqueous phase during the night a precipitate was formed which was collected by filtration. The crude products were crystallized from ethanol.

*4′,5′,6′-trimethoxy-[3,3′:3′,3″-terindolin]-2′-one* **4d**, yield 76%; ^1^H-NMR spectrum, δ, ppm: 2.86 (3H, s, OCH_3_), 3.65 (3H, s, OCH_3_), 3.84 (3H, s, OCH_3_), 6.47 (1H, s, is), 6.82 (2H, t, ind, J = 7.8), 6.84 (2H, d, ind-2, J = 2.0), 7.02 (2H, t, ind, J = 7.8), 7.27 (2H, d, ind, J = 7.8), 7.35 (2H, d, ind, J = 7.8), 10.41 (1H, s, NH, is), 10.89 (2H, d, NH, ind, J = 2.0). ^13^C-NMR spectrum, δ, ppm: 52.44, 55.96, 59.35, 60.55, 91.10, 111.47, 113.28, 117.55, 118.08, 120.69, 120.91, 125.12, 125.69, 136.63, 136.97, 137.52, 150.56, 153.70, 179.17. HRMS: *m*/*z* calcd. for C_27_H_23_N_3_O_4_ [M + Na]^+^: 476.1586; found: 476.1590. Anal. calcd. for C_27_H_23_N_3_O_4_ (MW 453.498): C, 71.51; H, 5.11; N, 9.27; found: C, 71.50; H, 5.13; N, 9.26.

*4′,6′-dimethyl-[3,3′:3′,3″-terindolin]-2′-one* **4e**, yield 83%; ^1^H-NMR spectrum, δ, ppm: 1.76 (3H, s, CH_3_), 2.30 (3H, s, CH_3_), 6.53 (1H, s, is), 6.69 (1H, s, is), 6.85 (2H, t, ind, J = 7.5), 6.87 (2H, s, ind), 7.05 (2H, t, ind, J = 7.5), 7.30 (2H, d, ind, J = 7.5), 7.39 (2H, d, ind, J = 7.5), 10.4 (1H, s, NH, is), 10.95 (2H, s, NH, ind). ^13^C-NMR spectrum, δ, ppm: 17.35, 21.20, 52.65, 79.19, 108.06, 11.57, 112.39, 118.17, 120.90, 120.97, 124.58, 125.19, 125.67, 128.35, 134.93, 136.87, 137.24, 141.82, 179.07. HRMS: *m*/*z* calcd. for C_26_H_21_N_3_O [M + Na]^+^: 414.1582; found: 414.1581. Anal. calcd. for C_26_H_21_N_3_O (MW 391.474): C, 79.77; H, 5.41; N, 10.73; found: C, 79.75; H, 5.42; N, 10.74.

*4′,7′-dichloro-[3,3′:3′,3″-terindolin]-2′-one* **4f**, yield 87%; ^1^H-NMR spectrum, δ, ppm: 6.88 (2H, d, ind, J = 7.7), 6.92 (2H, d, ind-2, J = 2.4), 6.97(1H, d, is, J = 8.2), 7.06 (2H, dt, ind, J = 7.7), 7.23 (2H, d, ind, J = 7.7), 7.39 (2H, d, ind, J = 7.7), 7.41 (1H, d, is, J = 8.2), 11.05 (2H, d, NH, ind, J = 2.4), 11.20 (1H, s, NH, is). ^13^C-NMR spectrum, δ, ppm: 54.46, 79.17, 109.84, 111.77, 112.88, 118.49, 120.32, 121.00, 124.04, 125.26, 126.01, 129.65, 129.76, 131.09, 136.74, 141.18, 177.83. HRMS: *m*/*z* calcd. for C_24_H_15_Cl_2_N_3_O [M + Na]^+^: 454.0490; found: 454.0485. Anal. calcd. for C_24_H_15_Cl_2_N_3_O (MW 432.304): C, 66.68; H, 3.50; N, 9.72; found: C, 66.68; H, 3.53; N, 9.69.

*5′-methoxy-1′-(4-methoxybenzyl)-[3,3′:3′,3″-terindolin]-2′-one* **4g**, yield 77%; ^1^H-NMR spectrum, δ, ppm: 3.61 (3H, s, OCH_3_), 3.73 (3H, s, OCH_3_), 4.91 (2H, s, CH_2_), 6.75 (2H, t, ind, J = 7.8), 6.84 (1H, dd, is-6, J = 8.6, J = 2.4), 6.86 (1H, d, is-4, J = 2.4), 6.88 (4H, m, 2 ind + 2 bn), 7.02 (2H, t, ind, J = 7.8), 7.03 (1H, d, is-7, J = 8.6), 7.13 (2H, d, ind, J = 7.8), 7.31 (2H, d, bn, J = 8), 7.36 (2H, d, ind, J = 7.8), 10.99 (2H, d, NH, ind, J = 2.0). ^13^C-NMR spectrum, δ, ppm: 42.53, 52.66, 55.06, 55.37, 109.71, 111.64, 111.80, 112.19, 113.90, 113.99, 118.23, 120.79, 121.00, 124.38, 125.59, 128.48, 129.09, 135.17, 135.18, 136.94, 155.19, 158.62, 176.67. HRMS: *m*/*z* calcd. for C_33_H_27_N_3_O_3_ [M + Na]^+^: 536.1950; found: 536.1947. Anal. calcd. for C_33_H_27_N_3_O_3_ (MW 513,597): C, 77.17; H, 5.30; N, 8.18; found: C, 77.20; H, 5.30; N, 8.15.

*4′-iodo-[3,3′:3′,3″-terindolin]-2′-one* **4h**, Yield 84%; ^1^H-NMR spectrum, δ, ppm: 6.85 (2H, t, ind, J = 7.9), 6.94 (2H, d, ind-2, J = 2.2), 7.05 (4H, m, 2 ind + 2 is), 7.23 (2H, d, ind, J = 7.9 Hz), 7.35 (1H, dd, is, J = 6, J = 2.8), 7.38 (2H, d, ind, J = 7.9), 10.65 (1H, s, NH, is), 11.01 (2H, d, NH, ind, J = 2.2). ^13^C-NMR spectrum, δ, ppm: 55.56, 94.74, 109.64, 109.96, 111.62, 118.29, 120.61, 120.85, 125.51, 127.22, 130.13, 132.58, 133.67, 136.73, 143.60, 178.27. HRMS: *m*/*z* calcd. for C_24_H_16_IN_3_O [M + Na]^+^: 512.0236; found: 512.0232. Anal. calcd. for C_24_H_16_IN_3_O (MW 489,316): C, 58.91; H, 3.30; N, 8.59; found: C, 58.94; H, 3.31; N, 8.55.

*5′-chloro-1′-methyl-[3,3′:3′,3″-terindolin]-2′-one* **4i**, yield 73%; ^1^H-NMR spectrum, δ, ppm: 3.24 (3H, s, CH_3_), 6.80 (2H, td, ind, J = 7.7, J = 1), 6.87 (2H, d, ind-2, J = 2.2), 7.02 (2H, td, ind, J = 7.7, J = 1), 7.12 (2H, d, ind, J = 7.7), 7.18 (1H, d, is-7, J = 8.1); 7.22 (1H, d, is-4, J = 2.3), 7.36 (2H, d, ind, J = 7.7), 7.39 (1H, dd, is-6, J = 8.1; J = 2.3), 11.02 (2H, d, NH, J = 2.2). ^13^C-NMR spectrum, δ, ppm: 26.44, 52.36, 110.32, 111.78, 113.28, 118.52, 120.44, 121.13, 124.40, 124.49, 125.43, 126.22, 127.94, 135.67, 136.96, 141.71, 176.53. HRMS: *m*/*z* calcd. for C_25_H_18_ClN_3_O [M + Na]^+^: 434.1036; found: 434.1035. Anal. calcd. for C_25_H_18_ClN_3_O (MW 411.889): C, 72.90; H, 4.41; N, 10.20; found: C, 72.87; H, 4.40; N, 10.24.

*6′-(trifluoromethoxy)-[3,3′:3′,3″-terindolin]-2′-one* **4l**, yield 87%; ^1^H-NMR spectrum, δ, ppm: 6.80 (2H, t, ind, J = 7.9), 6.86 (2H, d, ind-2, J = 2.2), 6.90 (2H, m, is), 7.02 (2H, t, ind, J = 7.9), 7.20 (2H, d, ind, J = 7.9), 7.32 (1H, d, is, J = 8.4), 7.35 (2H, d, ind, J = 7.9), 10.80 (1H, s, NH, is), 11.00 (2H, s, NH, ind). ^13^C-NMR spectrum, δ, ppm: 25.47, 52.27, 62.01, 102.64, 111.69, 113.60, 118.36, 120.51, 121.03, 124.30, 125.53, 126.16, 133.58, 136.94, 142.77, 147.84, 178.66. HRMS: *m*/*z* Calcd. for C_25_H_16_F_3_N_3_O_2_ [M + Na]^+^: 470.1092; found: 470.1088. Anal. calcd. for C_25_H_16_F_3_N_3_O_2_ (MW 447.417): C, 67.11; H, 3.60; N, 9.39; found: C, 67.14; H, 3.58; N, 9.38.

*1′-(4-chlorobenzyl)-[3,3′:3′,3″-terindolin]-2′-one* **4m**, yield 81%; ^1^H-NMR spectrum, δ, ppm: 5.01 (2H, s, CH_2_), 6.73 (2H, t, ind, J = 7.6), 6.86 (2H, d, ind-2, J = 2.4), 7.00 (3H, m, is + 2 ind), 7.09 (2H, d, bn, J = 8.2), 7.15 (1H, d, is, J = 7.4), 7.27 (1H, t, is, J = 7.4), 7.30 (1H, d, is, J = 7.4), 7.35 (2H, d, bn, J = 8.2), 7.39 (4H, m, ind), 11.00 (2H, d, NH, ind, J = 2.4). ^13^C-NMR spectrum, δ, ppm: 42.36, 52.25, 109.26, 111.66, 113.9, 118.22, 120.69, 121.01, 122.39, 124.34, 124.84, 125.56, 127.92, 128.56, 129.61, 132.15, 133.77, 135.65, 136.94, 141.53, 177.07. HRMS: *m*/*z* calcd. for C_31_H_22_ClN_3_O [M + Na]^+^: 510.1349; found: 510.1353. Anal. calcd. for C_31_H_22_ClN_3_O (MW 487.987): C, 76.30; H, 4.54; N, 8.61; found: C, 76.34; H, 4.51; N, 8.60.

*4′,5,5′,5″,6′-pentamethoxy-[3,3′:3′,3″-terindolin]-2′-one* **5d**, Yield 73%; ^1^H-NMR spectrum, δ, ppm: 2.86 (3H, s, OCH_3_, is), 3.56 (6H, s, OCH_3_, ind), 3.65 (3H, s, OCH_3_, is), 3.83 (3H, s, OCH_3_, is), 6.48 (1H, s, is-7), 6.70 (2H, d, ind-6, J = 8.8), 6.74 (2H, s, ind-2), 6.86 (2H, s, ind-4), 7.25 (2H, d, ind-7, J = 8.8), 10.42 (1H, s, NH, is), 10.74 (2H, s, NH, ind). ^13^C-NMR spectrum, δ, ppm: 52.38, 55.17, 56.05, 59.27, 60.55, 91.01, 103.46, 110.19, 111.89, 112.66, 117.58, 125.91, 126.11, 131.89, 137.03, 137.66, 150.57, 152.35, 153.75, 179.14. HRMS: *m*/*z* calcd. for C_29_H_27_N_3_O_6_ [M + Na]^+^: 536.1798; found: 536.1794. Anal. calcd. for C_29_H_27_N_3_O_6_ (MW 513.550): C, 67.83; H, 5.30; N, 8.18; found: C, 67.80; H, 5.31; N, 8.20.

*1′-(4-chlorobenzyl)-5,5″-dimethoxy-[3,3′:3′,3″-terindolin]-2′-one* **5m**, yield 93%; ^1^H-NMR spectrum, δ, ppm: 3.42 (6H, s, OCH_3_), 5.01 (2H, s, CH_2_), 6.54 (2H, d, ind-4, J = 2.5), (2H, dd, ind-6, J = 8.6, J = 2.5), 6.88 (2H, d, ind-2, J = 2.4), 7.02 (1H, t, is, J = 7.6), 7.15 (1H, d, is, J = 7.6), 7.27 (4H, m, 2 ind + 2 is), 7.35 (2H, d, bn, J = 8.4), 7.40 (2H, d, bn, J = 8.4), 10.85 (2H, d, NH, J = 2.4). ^13^C-NMR spectrum, δ, ppm: 42.34, 52.22, 54.92, 102.95, 109.10, 110.69, 112.20, 113.18, 122.47, 124.90, 125.24, 125.93, 127.89, 128.57, 129.56, 132.14, 133.73, 135.77, 141.63, 152.45, 177.15. HRMS: *m*/*z* calcd. for C_33_H_26_ClN_3_O_3_ [M + Na]^+^: 570.1560; found: 570.1561. Anal. calcd. for C_33_H_26_ClN_3_O_3_ (MW 548.039): C, 72.32; H, 4.78; N, 7.67; found: C, 72.33; H, 4.79; N, 7.65.

*3,3-bis(6-chloroimidazo[2,1-*b*]thiazol-5-yl)indolin-2-one* **6c**, yield 68%; ^1^H-NMR spectrum, δ, ppm: 6.88 (1H, d, th, J = 4.7), 7.02 (2H, m, is), 7.13 (1H, d, th, J = 4.7), 7.23 (1H, d, is, J = 7.2), 7.28 (1H, d, th, J = 4.7), 7.35 (2H, m, th + is), 11.20 (1H, s, NH). ^13^C-NMR spectrum, δ, ppm: 50.45, 110.81, 114.59, 114.98, 115.67, 115.78, 119.20, 119.41, 122.53, 126.09, 126.75, 130.27, 130.35, 131.13, 141.38, 147.69, 147.84, 172.72. HRMS: *m*/*z* Calcd. for C_18_H_9_Cl_2_N_5_OS_2_ [M + H]^+^: 445.9704; found: 445.9688. Anal. calcd. for C_18_H_9_Cl_2_N_5_OS_2_ (MW 446.324): C, 48.44; H, 2.03; N, 15.69; found: C, 48.45; H, 2.01; N, 15.70.

*3,3-bis(6-chloroimidazo[2,1-*b*]thiazol-5-yl)-5-methoxy-1-(4-methoxybenzyl)indolin-2-one* **6g**, Yield 74%; ^1^H-NMR spectrum, δ, ppm: 3.65 (3H, s, OCH_3_), 3.72 (3H, s, OCH_3_), 4.91 (1H, d, CH_2,_ J = 15.4), 4.98 (1H, d, CH_2,_ J = 15.4), 6.88 (4H, m, th + is + 2 bn), 6.94 (1H, d, th, J = 4.4), 6.98 (1H, dd, is-6, J = 8.8, J = 2.6), 7.18 (1H, d, is-7, J = 8.8), 7.26 (4H, m, 2 th + 2 bn). ^13^C-NMR spectrum, δ, ppm: 43.19, 50.29, 55.09, 55.59, 111.19, 112.92, 113.99, 114.50, 114.70, 114.84, 115.37, 115.53, 119.18, 119.37, 127.25, 127.39, 128.94, 130.43, 131.18, 135.26, 147.86, 147.94, 155.76, 158.77, 170.74. HRMS: *m*/*z* Calcd. for C_27_H_19_Cl_2_N_5_O_3_S_2_ [M + H]^+^: 596.0385; found: 596.0385. Anal. calcd. for C_27_H_19_Cl_2_N_5_O_3_S_2_ (MW 596.501): C, 54.37; H, 3.21; N, 11.74; found: C, 54.38; H, 3.20; N, 11.74.

*3,3-bis(6-chloroimidazo[2,1-*b*]thiazol-5-yl)-5-methoxyindolin-2-one* **6k**, Yield 77%; ^1^H-NMR spectrum, δ, ppm: 3.65 (3H, s, OCH_3_), 6.82 (1H, d, is-4, J = 1.6), 6.90 (1H, d, th, J = 4.4), 6.95 (2H, m, is-6 + is-7), 7.15 (1H, d, th, J = 4.4), 7.27 (1H, d, th, J = 4.4), 7.34 (1H, d, th, J = 4.4), 11.04 (1H, s, NH).^13^C-NMR spectrum, δ, ppm: 50.80, 55.53, 111.34, 112.83, 114.49, 114.88, 114.93, 115.72, 115.76, 119.23, 119.51, 127.95, 130.39, 131.08, 134.64, 147.67, 147.78, 155,18, 172.51. HRMS: *m/z* Calcd. for C_19_H_11_Cl_2_N_5_O_2_S_2_ [M + H]^+^: 475.9809; found: 596. 475.9801. Anal. calcd. for C_19_H_11_Cl_2_N_5_O_2_S_2_ (MW 476.350): C, 47.91; H, 2.33; N, 14.70; found: C, 47.94; H, 2.29; N, 14.71.

*1-(4-chlorobenzyl)-3,3-bis(6-chloroimidazo[2,1-*b*]thiazol-5-yl)indolin-2-one* **6m**, yield 81%;^1^H-NMR spectrum, δ, ppm: 5.02 (1H, d, CH_2,_ J = 16.0), 5.08 (1H, d, CH_2,_ J = 16.0), 6.84 (1H, d, th, J = 4.6), 7.00 (1H, d, th, J = 4.6), 7.09 (1H, t, is, J = 7.7), 7.25 6.94 (1H, d, is, J = 7.7), 7.35 (8H, m, 2 th + 2 is + 4 ar). ^13^C-NMR spectrum, δ, ppm: 42.99, 49.96, 110.47, 114.93, 114.98, 115.04, 115.59, 119.07, 119.21, 123.45, 125.80, 126.05, 128.62, 129.37, 130.33, 130.35, 131.20, 132.37, 134.61, 141.77, 147.90, 148.06, 171.12. HRMS: *m*/*z* calcd. for C_25_H_14_Cl_3_N_5_OS_2_ [M + H]^+^: 569.9784; found: 569.9783. Anal. calcd. for C_25_H_14_Cl_3_N_5_OS_2_ (MW 570.891): C, 52.60; H, 2.47; N, 12.27; found: C, 52.61; H, 2.48; N, 12.25

### 3.2. In Vitro Susceptibility Testing

The in vitro antimicrobial activity of the isatin derivatives was evaluated by a microdilution broth method in accordance with the guidelines provided by a number of international committees, such as The Clinical Laboratory Standards Institute (CLSI) in the U.S. or The European Committee on Antimicrobial Susceptibility Testing (EUCAST) in Europe reference bacterial strains, including *Staphylococcus aureus* (ATCC 25923) and *Escherichia coli* (ATCC 25922) and *Candida albicans* (ATCC 10231), were purchased from the American Type Culture Collection (ATCC); they were routinely cultured in 5% sheep blood agar plate and in Sabauraud dextrose agar, respectively, at 37 °C. Clinical isolates, identified by MALDI-TOF MS (Bruker Daltonik, GmbH, Bremen, Germany), were profiled for their antibiotic susceptibility by using the Vitek2 semiautomated system (bioMerieux, Craponne, France) and according to EUCAST guidelines [EUCAST: The European Committee on Antimicrobial Susceptibility Testing. Breakpoint tables for interpretation of MICs and zone diameters. Version 12, 2022, http://www.eucast.org (accessed on 1 January 2022)].

Microbial inocula were prepared at 0.5 McFarland in PBS and, subsequently, bacterial suspensions were diluted 1:200 in Mueller–Hinton broth (Sigma-Aldrich, St. Louis, MO, USA), while fungal inoculum was diluted 1:20 in RPMI-1640 medium (Gibco^®^, ThermoFisher Scientific Inc., Waltham, MA, USA), containing glucose 2%, 0.3% levo-glutamine buffered to pH 7.0 with 0.165 M 3-(N-morpholino)propanesulfonic acid (MOPS). A total of 100 µL of these microbial suspensions were introduced in a 96-well microplate and treated with 100 µL of the compound at 100 µM or with two-fold serial dilutions of the compound in the range of 400 µM–3.12 µM. All compounds were previously dissolved in 100% DMSO at a concentration of 20 mM. Experiments included controls used to measure the microbial growth in regular medium (positive control) and to check the background turbidity of the reagents and the sterility of the procedures (negative controls). In addition, microbial growths were assessed in the presence of the DMSO solvent in the range of 0.015–2%. The inoculated plate was incubated at 37 °C for 24 h, and subsequently, the optical density at 630 nm was measured by the Multiskan Ascent microplate reader (Thermo Fisher Scientific Inc., Waltham, MA, USA). The effectiveness of the compounds was expressed as percent inhibition relative to the positive growth controls, and the isatin derivatives were defined as active when the inhibition yielded the 50% at 100 µM. At the tested concentrations, the DMSO did not interfere with cell proliferations. These compounds were further evaluated to define their IC_50_ values by interpolation of the dose–response curves generated by plotting the percentages of growth inhibition, relative to the positive control (set to 100% of growth), as a function of the tested concentrations (GraphPad Prism version 5.0 for Windows, San Diego, CA, USA). All the experiments were performed on triplicate in at least two independent assays.

### 3.3. Cytotoxicity Test

African green monkey kidney cells (Vero ATCC CCL-81) obtained from ATCC were cultured in Eagle’s minimal essential medium (MEM) (Sigma-Aldrich, St. Louis, MO, USA), supplemented with 10% fetal bovine serum (FBS) (Carlo Erba Reagents, Milan, Italy), 100 U/mL penicillin, and 100 µg/mL streptomycin at 37 °C with 5% CO_2_. For the experiments, the cells were seeded into 96-well plates at 10^4^ cells/well and incubated at 37 °C for 24 h. Following washes with PBS, the cell monolayer was incubated with 100 µL of medium containing 100 µM of the derivatives. Both untreated cells and cells incubated with the solvent were included in each experiment as controls.

The cell viability was assessed by a WST8-based assay according to the manufacturer’s instructions (CCK-8, Cell Counting Kit-8, Dojindo Molecular Technologies, Rockville, MD, USA). After 48 h of incubation, the culture medium was removed from each well, the monolayer was washed with PBS, and 100 µL of fresh medium containing 10 µL of CCK-8 solution were added. Following 2 h at 37 °C, the absorbance was measured at 450/630 nm; data were calculated as the percentage of the cell viability relative to the untreated controls. At the tested concentrations, the DMSO did not interfere with Vero metabolism. Isatin derivatives demonstrating inhibitory activity on Vero cells metabolism superior to 70% at 100 µM were defined as cytotoxic. All the experiments were performed on triplicate in at least two independent assays.

## 4. Conclusions

A small library of 18 isatin-based derivatives was synthetized and evaluated for their antimicrobial potential on three reference strains, including *S. aureus*, *E. coli*, and *C. albicans*. The study delivered two lead compounds, **6k** and **6m**, endowed with excellent inhibitory activity against *S. aureus* in comparison with other isatin hybrids reported in literature. The compounds were found to be sufficiently soluble for the herein performed in vitro tests (as reported in the section Materials and Methods); however, they are not very soluble as determined by in silico analysis, using the free online software SwissADME (http://www.swissadme.ch/index.php (accessed on 25 July 2022). From a future perspective, in the next design we will consider the solubility improvement based on the structure of the most interesting ones. The described isatin derivatives showed good antibacterial activity on the reference strain as well as on 10 clinical isolates obtained from different biological specimens indicating that the compounds have in vitro potential also towards human pathogens circulating in the population. As the isatin derivatives revealed a fascinating array of pharmacological activities, including broad-spectrum antiproliferative properties, the herein synthetized molecules were also evaluated for their cytotoxicity on mammalian cells, and the selective inhibitory effect towards bacterial cell was confirmed for **6k** and **6m**. The overall data therefore indicate the importance of the isatin nucleus in the field of medicinal chemistry as an antibacterial agent. From a future perspective, derivatives demonstrating cytotoxicity on the non-malignant cell model herein used could be investigated on cancer cell lines to measure their anti-cancer potency.

## Figures and Tables

**Table 1:** New derivatives.

Comp.	R	R_1_	R_2_	R_3_	R_4_	R_5_
**4c** [18]	H	H	H	H	H	H
**4d**	H	H	OCH_3_	OCH_3_	OCH_3_	H
**4e**	H	H	CH_3_	H	CH_3_	H
**4f**	H	H	Cl	H	H	Cl
**4g**	H	4-MBn	H	OCH_3_	H	H
**4h**	H	H	I	H	H	H
**4i**	H	CH_3_	H	Cl	H	H
**4j** [19]	H	H	F	H	H	H
**4k** [19]	H	H	OCH_3_	H	H	H
**4l**	H	H	H	H	OCF_3_	H
**4m**	H	4-ClBn	H	H	H	H
**5d**	OCH_3_	H	OCH_3_	OCH_3_	OCH_3_	H
**5k** [19]	OCH_3_	H	H	OCH_3_	H	H
**5m**	OCH_3_	4-ClBn	H	H	H	H
**6c**		H	H	H	H	H
**6g**		4-MBn	H	OCH_3_	H	H
**6k**		H	H	OCH_3_	H	H
**6m**		4-ClBn	H	H	H	H

4-MBn = 4-methoxybenzyl, 4-ClBn = 4-chlorobenzyl.

**Table 2:** Microbial growth and cell proliferation (mean values and standard deviations).

Comp.	*S. aureus*	*E. coli*	*C. albicans*	Vero
**4c**	1.3 ± 1.5	111.2 ± 1.5	106.8 ± 5.0	9.9 ± 1.3
**4d**	84.3 ± 5.8	116.4 ± 0.6	120.8 ± 7.6	41.2 ± 0.1
**4e**	71.2 ± 8.7	103.3 ± 1.4	68.8 ± 2.7	8.5 ± 0.3
**4f**	75.8 ± 1.8	96.9 ± 1.7	106.7 ± 4.2	9.8 ± 0.5
**4g**	86.2 ± 5.7	100.3 ± 0.7	50.6 ± 6.6	107.9 ± 8.9
**4h**	72.8 ± 6.1	82.0 ± 1.5	23.2 ±2.1	10.6 ± 0.3
**4i**	64.2 ± 4.3	103.6 ± 1.2	51.7 ± 3.9	69.7 ± 8.6
**4j**	66.4 ± 9.9	98.1 ± 3.0	77.8 ± 10.5	9.2 ± 0.3
**4k**	62.2 ± 4.1	110.5 ± 1.1	67.8 ± 3.7	9.4 ± 0.9
**4l**	72.0 ± 2.2	103.2 ± 1.6	93.3 ± 2.1	8.9 ±0.4
**4m**	77.9 ± 6.7	107.2 ± 1.2	106.1 ± 2.7	58.2 ± 5.9
**5d**	89.1 ± 7.0	117.0 ± 1.1	120.0 ± 2.0	54.3 ± 0.9
**5k**	76.0 ± 5.0	113.3 ± 1.1	66.0 ± 5.2	99.6 ± 6.8
**5m**	95.1 ± 3.9	113.0 ± 0.6	88.2 ± 5.2	87.0 ± 5.7
**6c**	95.4 ± 2.4	117.3 ± 3.1	126.3 ± 2.1	92.8 ± 4.8
**6g**	67.4 ± 8.4	73.6 ± 9.1	92.4 ± 3.6	84.3 ± 0.9
**6k**	43.4 ± 5.6	102.7 ± 2.4	104.1 ± 1.9	70.9 ± 5.6
**6m**	48.1 ± 11.4	88.6 ± 6.2	81.5 ± 2.0	90.7 ± 1.2

## Data Availability

The data presented in this study are available in this article.

## Supplementary Materials

The supporting information can be download at https://www.mdpi.com/article/10.3390/molecules27185781/s1, ^1^H NMR and ^13^C NMR spectra; HRMS spectra; Figure S1: Microbial growth and cell proliferation; Table S1: Antibiotic resistance profile.

## Sample Availability

Samples of the compounds are available from the corresponding author.
