# Supplementary material for: Isatin Bis-Indole and Bis-Imidazothiazole Hybrids: Synthesis and Antimicrobial Activity
Source: Molecules. 2022 Sep 7;27(18):5781. doi: 10.3390/molecules27185781 (PMC9505023; doi:10.3390/molecules27185781)
Supplement: Supplementary file 1 [file molecules-27-05781-s001.zip › molecules-1857610-supplementary.pdf]

Bis-indole and bis-imidazothiazole isatin hybrids: synthesis and antimicrobial activity.

Francesca Bonvicini [1], Alessandra Locatelli [2,*], Rita Morigi [2], Alberto Leoni [2,†] and Giovanna Angela Gentilomi [1,3,†]

[1] Department of Pharmacy and Biotechnology, Alma Mater Studiorum-University of Bologna, Via Massarenti 9, 40138 Bologna, Italy

[2] Department of Pharmacy and Biotechnology, Alma Mater Studiorum-University of Bologna, Via Belmeloro 6, 40126 Bologna, Italy

[3] Division of Microbiology, IRCCS Azienda Ospedaliero-Universitaria di Bologna, Via Massarenti 9, 40138 Bologna, Italy

* Correspondence: alessandra.locatelli@unibo.it; Tel.: +39-0512099712

† These authors contributed equally to this work.

Contents:

^{1}H NMR and ^{13}C NMR spectra……………………..……………………………pag. 2-15

HRMS spectra……………………………………………………………………pag. 16-20

Figure S1 (Microbial growth and cell proliferation) ……………………………pag. 21

Table S1 (Antibiotic resistance profile) ……………………………………….pag. 22

^{1}H and ^{13}C NMR spectra of the described compounds

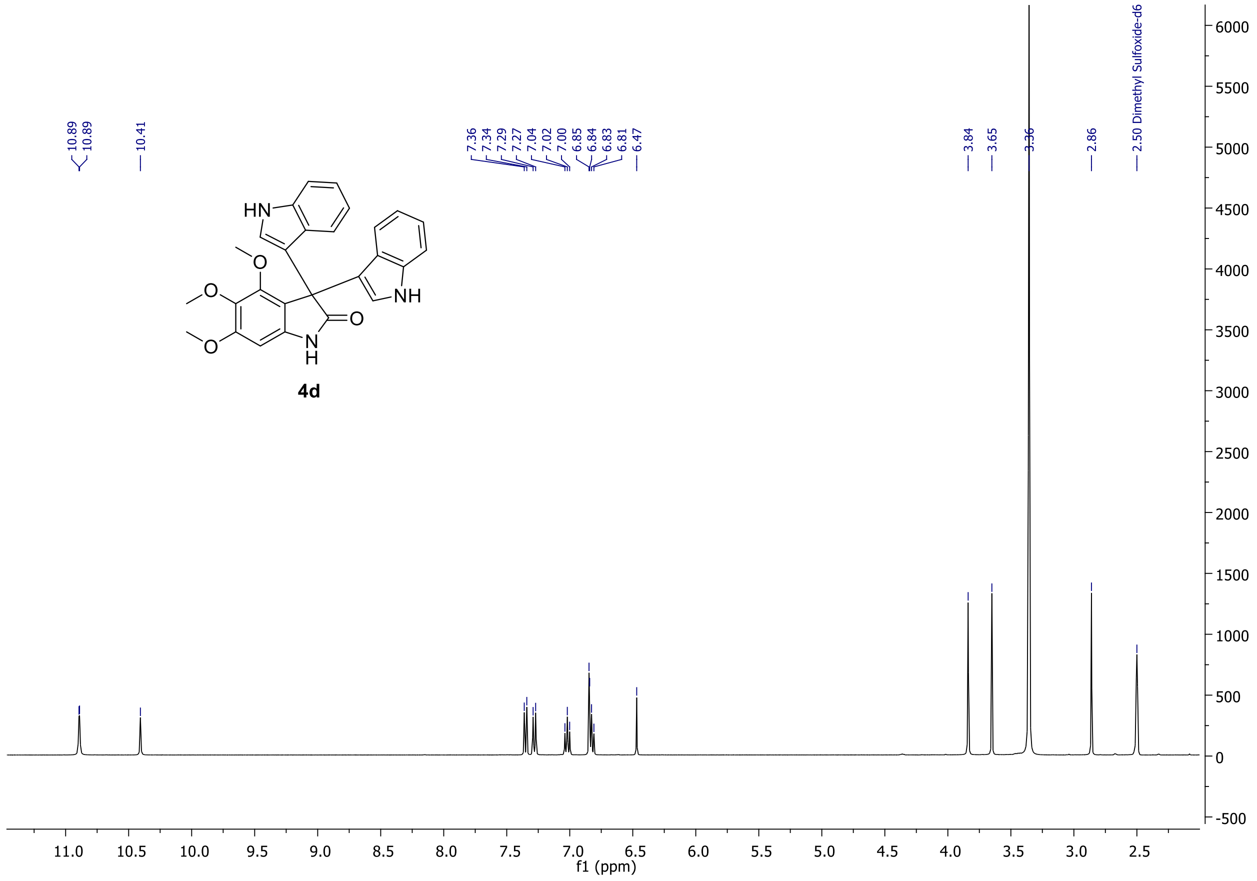

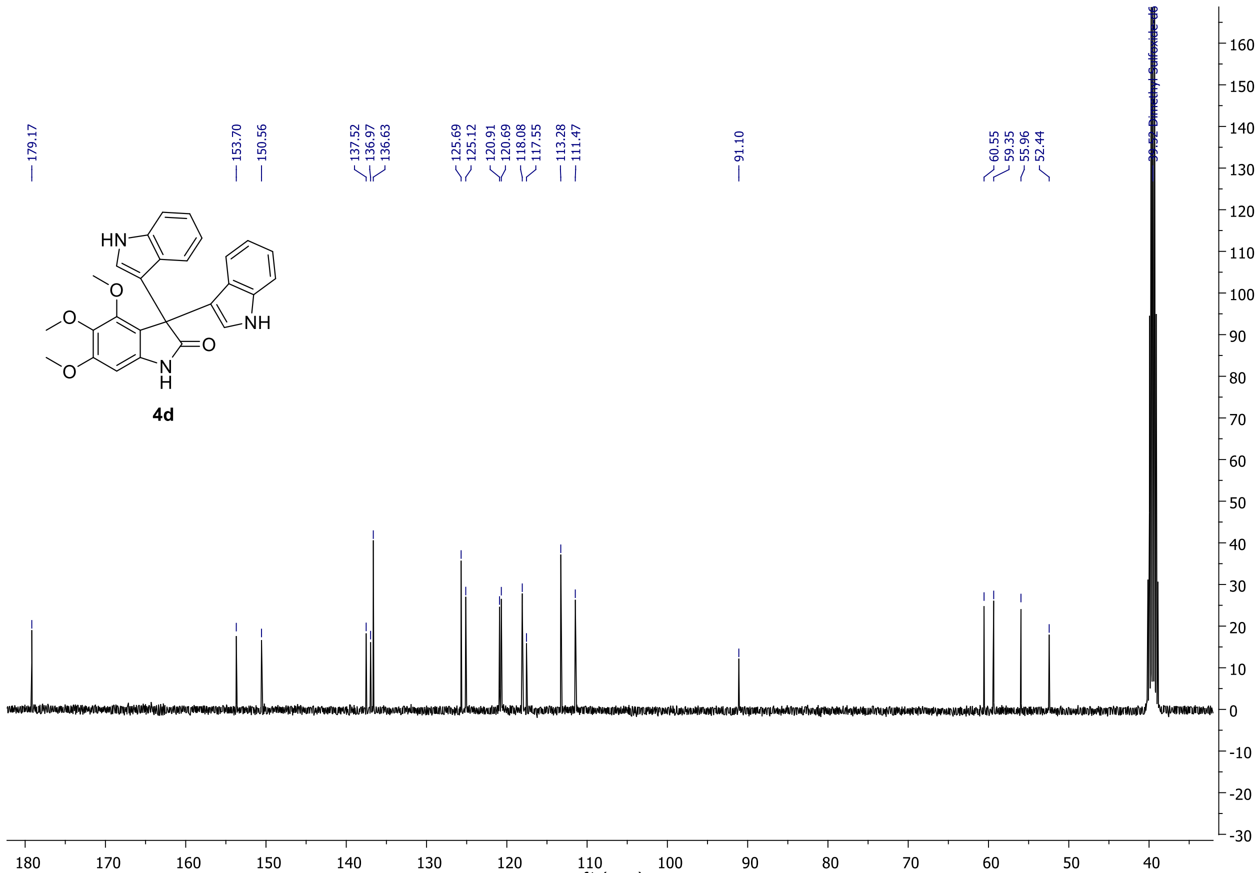

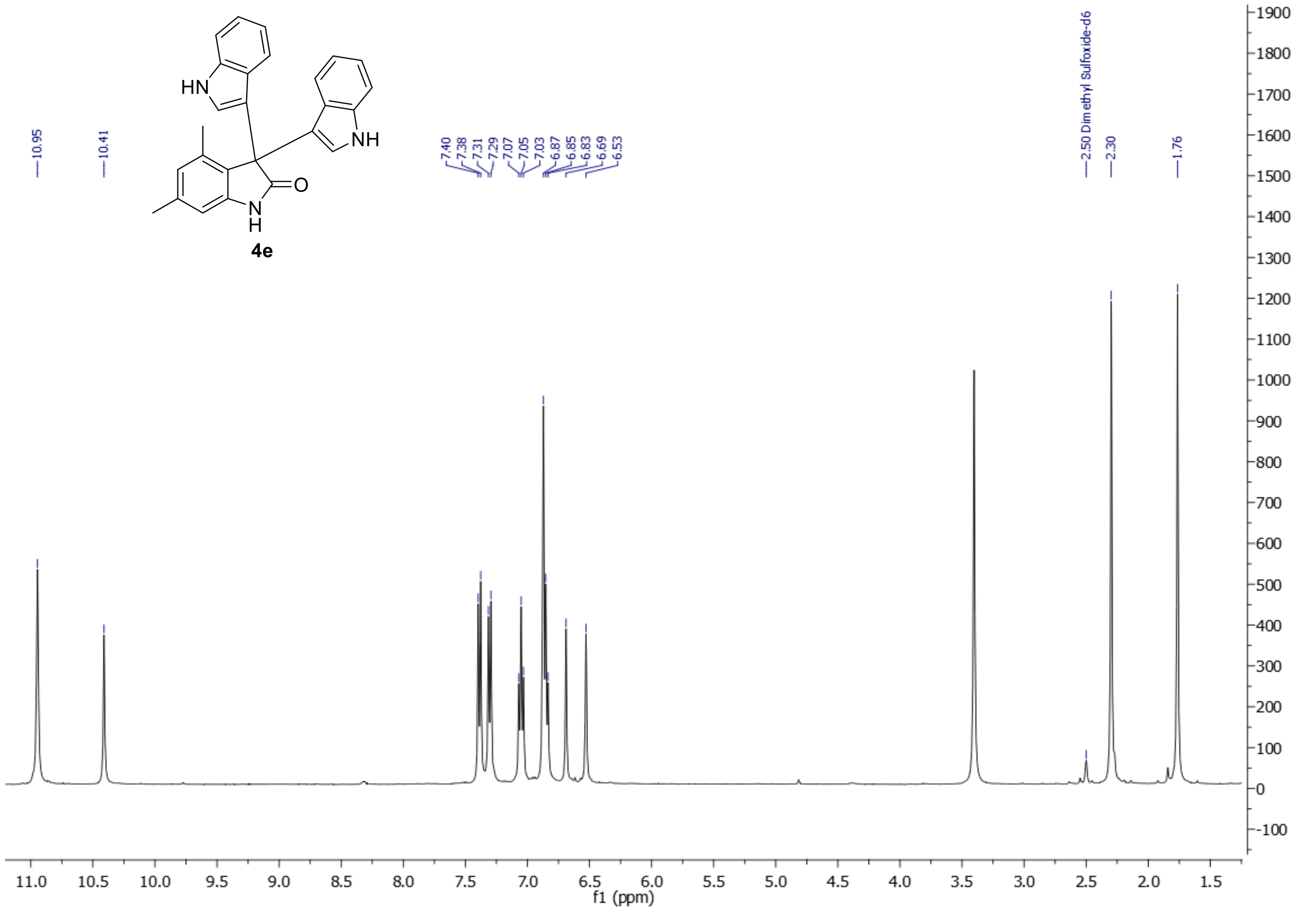
10.95
10.41
7.40
7.38
7.31
7.29
7.07
7.05
7.03
6.87
6.85
6.83
6.69
6.53
2.50 Dimethyl Sulfoxide-d6
2.30
1.76
HN
NH
O
N
H
4e
f1 (ppm)

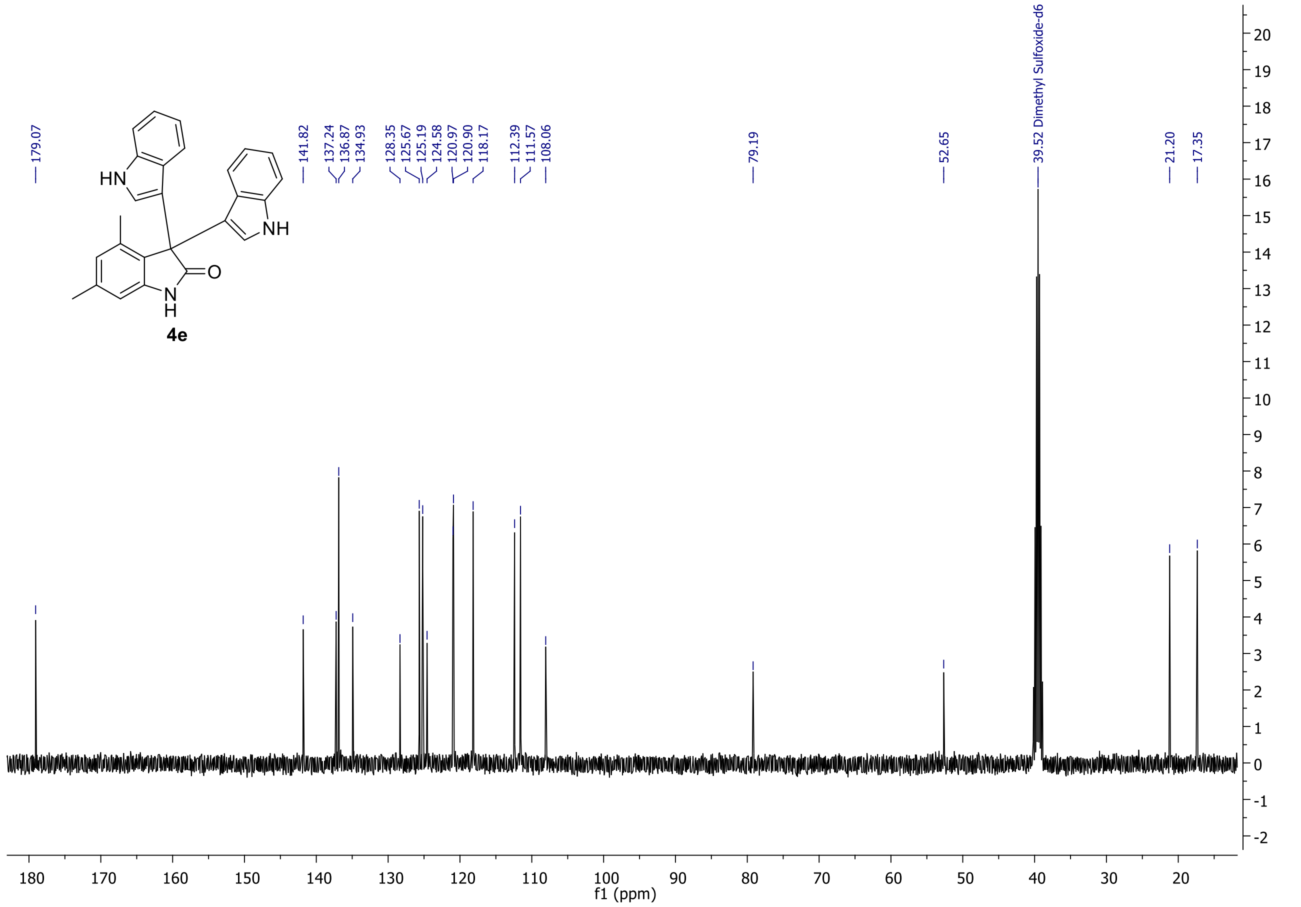
179.07
141.82
137.24
136.87
134.93
128.35
125.67
125.19
124.58
120.97
120.90
118.17
112.39
111.57
108.06
79.19
52.65
39.52 Dimethyl Sulfoxide-d6
21.20
17.35
HN
NH
O
N
H
4e
f1 (ppm)

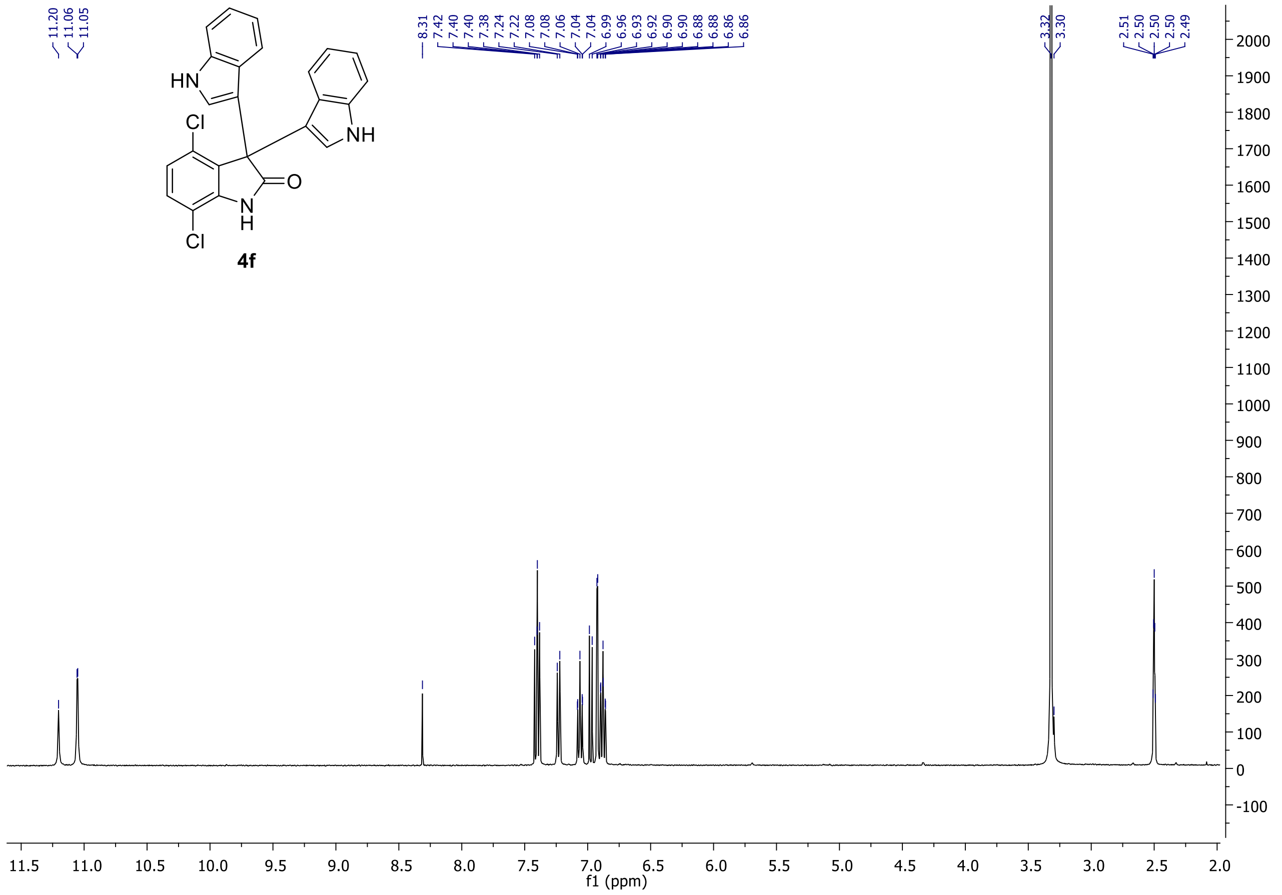
11.20
11.06
11.05
8.31
7.42
7.40
7.40
7.38
7.24
7.22
7.08
7.08
7.06
7.04
7.04
6.99
6.96
6.93
6.92
6.90
6.90
6.88
6.86
6.86
3.32
3.30
2.51
2.50
2.50
2.50
2.49
HN
Cl
NH
O
N
H
Cl
4f
f1 (ppm)

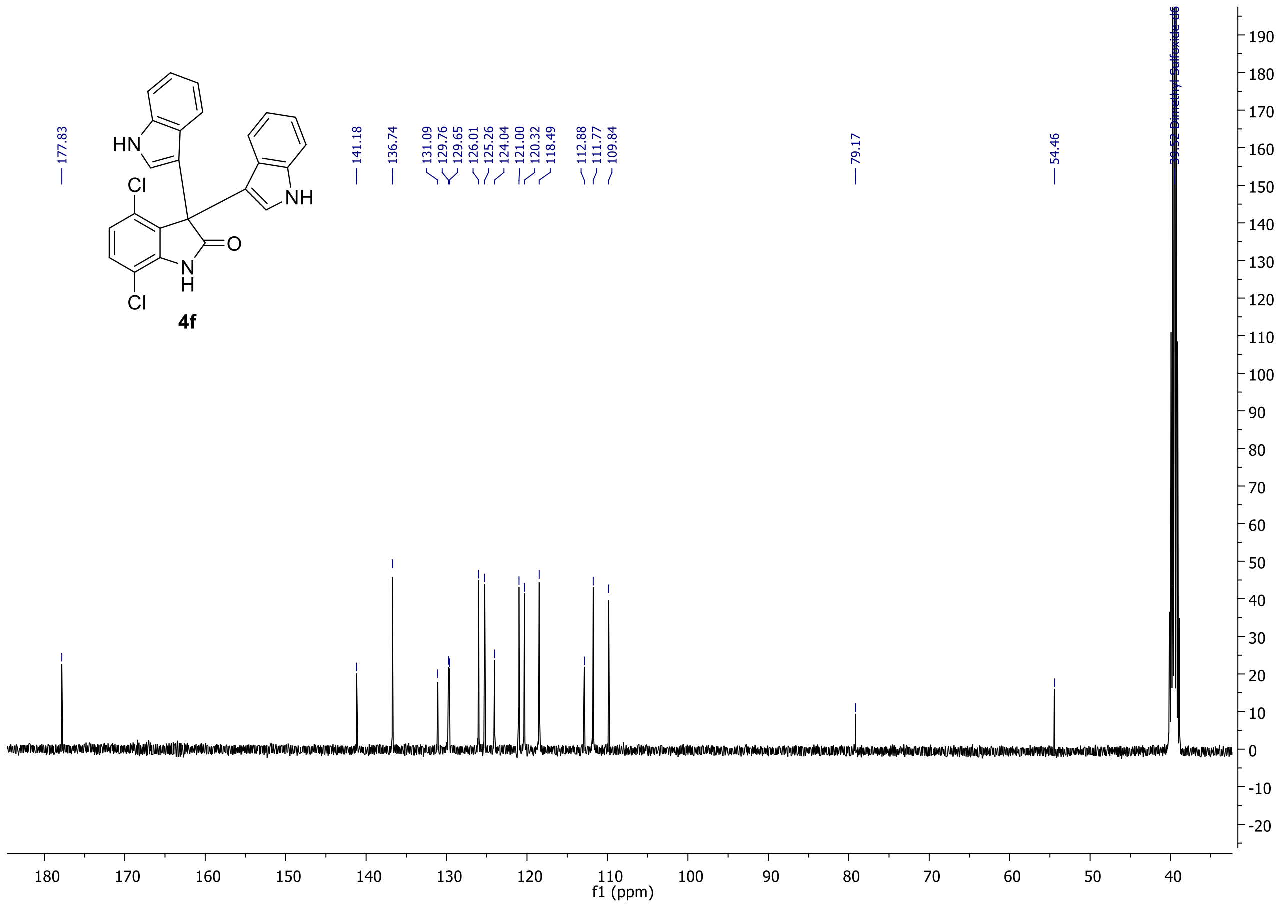
177.83
141.18
136.74
131.09
129.76
129.65
126.01
125.26
124.04
121.00
120.32
118.49
112.88
111.77
109.84
79.17
54.46
HN
Cl
NH
O
N
H
Cl
4f
f1 (ppm)

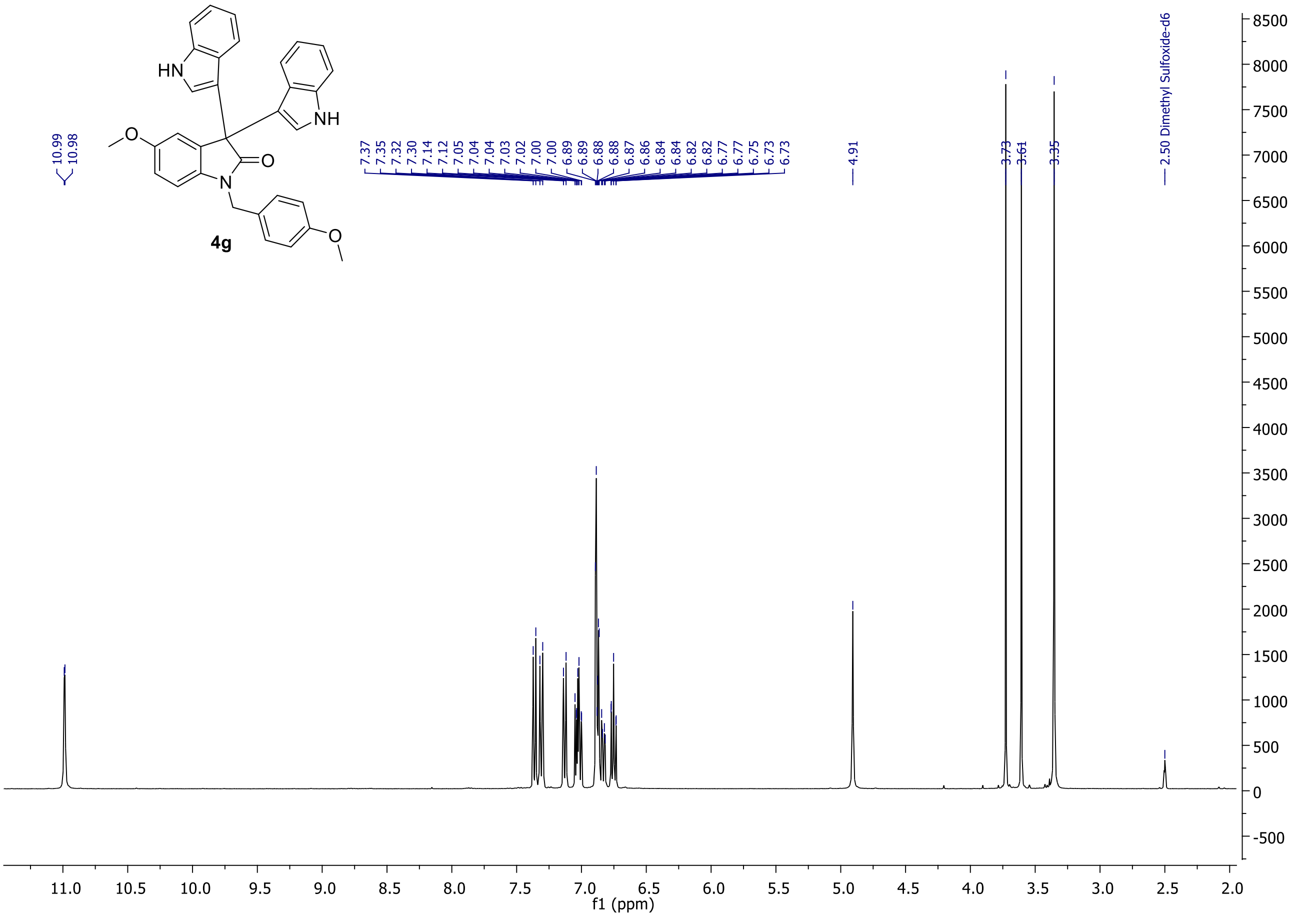

HN
NH
O
N
4g
10.99
10.98
7.37 7.35 7.32 7.30 7.14 7.12 7.05 7.04 7.04 7.03 7.02 7.00 6.89 6.89 6.88 6.88 6.87 6.86 6.84 6.84 6.82 6.82 6.77 6.77 6.75 6.73 6.73
4.91
3.73
3.61
3.35
2.50 Dimethyl Sulfoxide-d6
f1 (ppm)

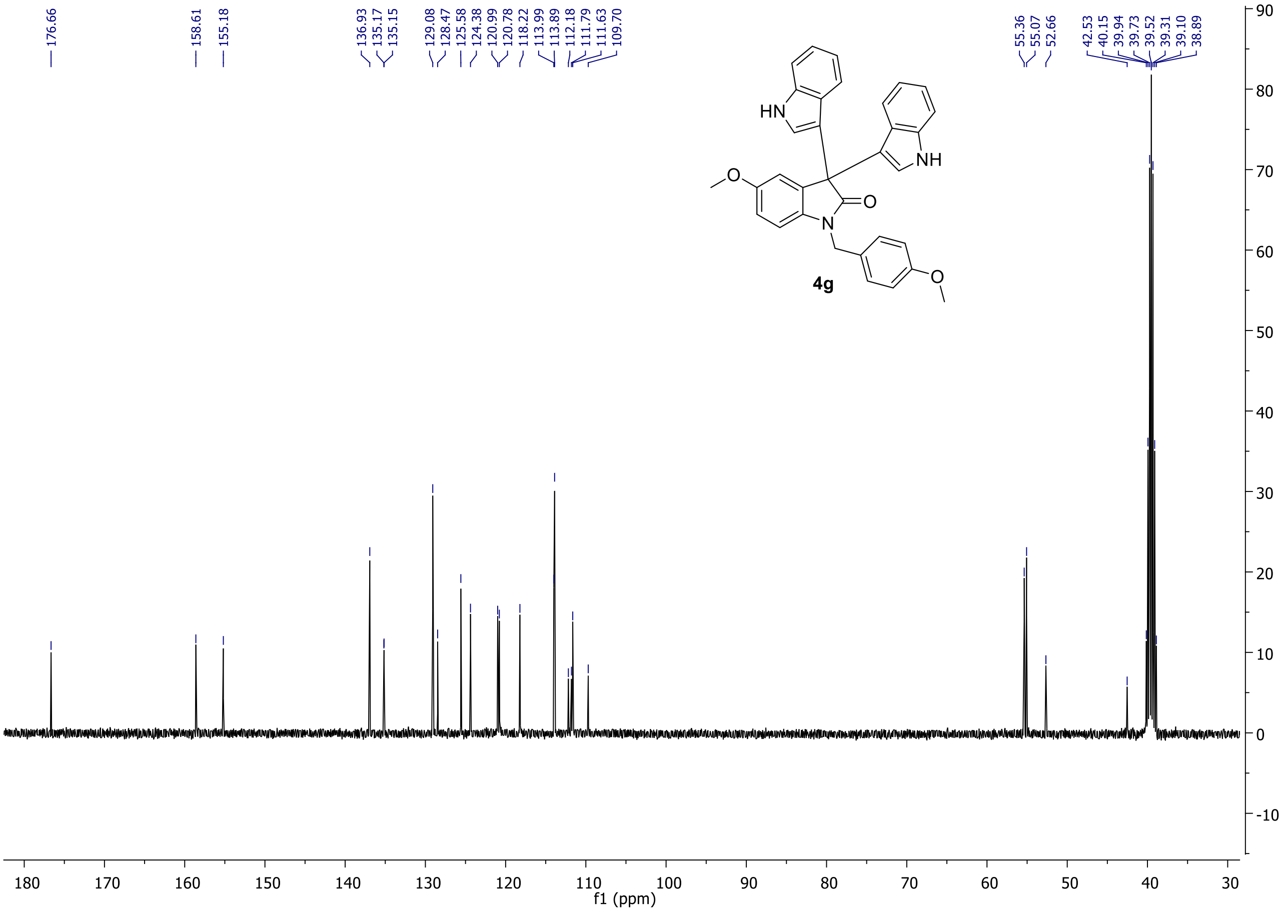

176.66
158.61
155.18
136.93 135.17 135.15
129.08 128.47 125.58 124.38 120.99 120.78 118.22 113.99 113.89 112.18 111.79 111.63 109.70
55.36 55.07 52.66
42.53 40.15 39.94 39.73 39.52 39.31 39.10 38.89
HN
NH
O
N
4g
f1 (ppm)

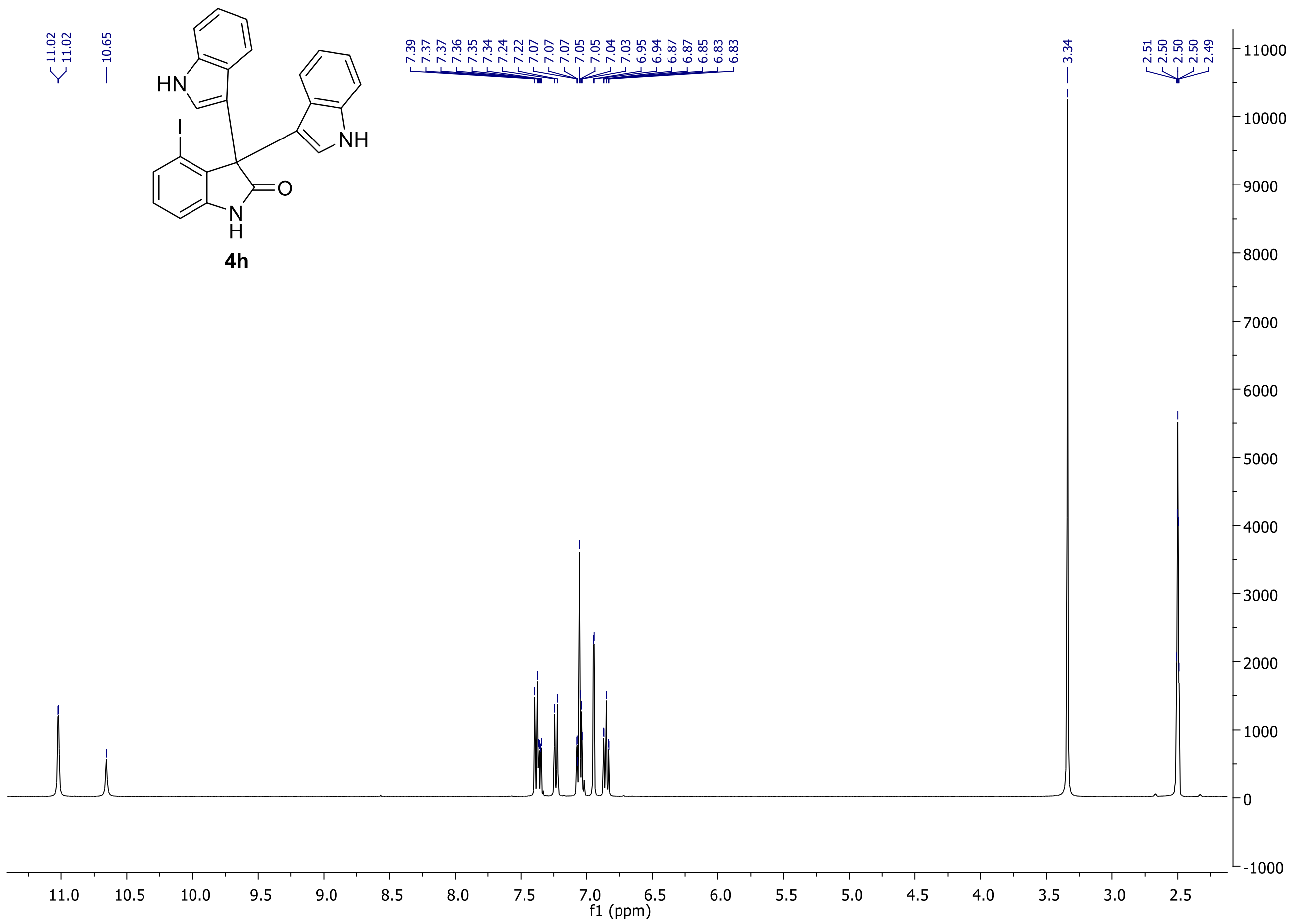
11.02
11.02
10.65
7.39
7.37
7.37
7.36
7.35
7.34
7.24
7.22
7.07
7.07
7.07
7.05
7.05
7.04
7.03
6.95
6.94
6.87
6.87
6.85
6.83
6.83
3.34
2.51
2.50
2.50
2.50
2.49
HN
NH
I
O
N
H
4h
f1 (ppm)

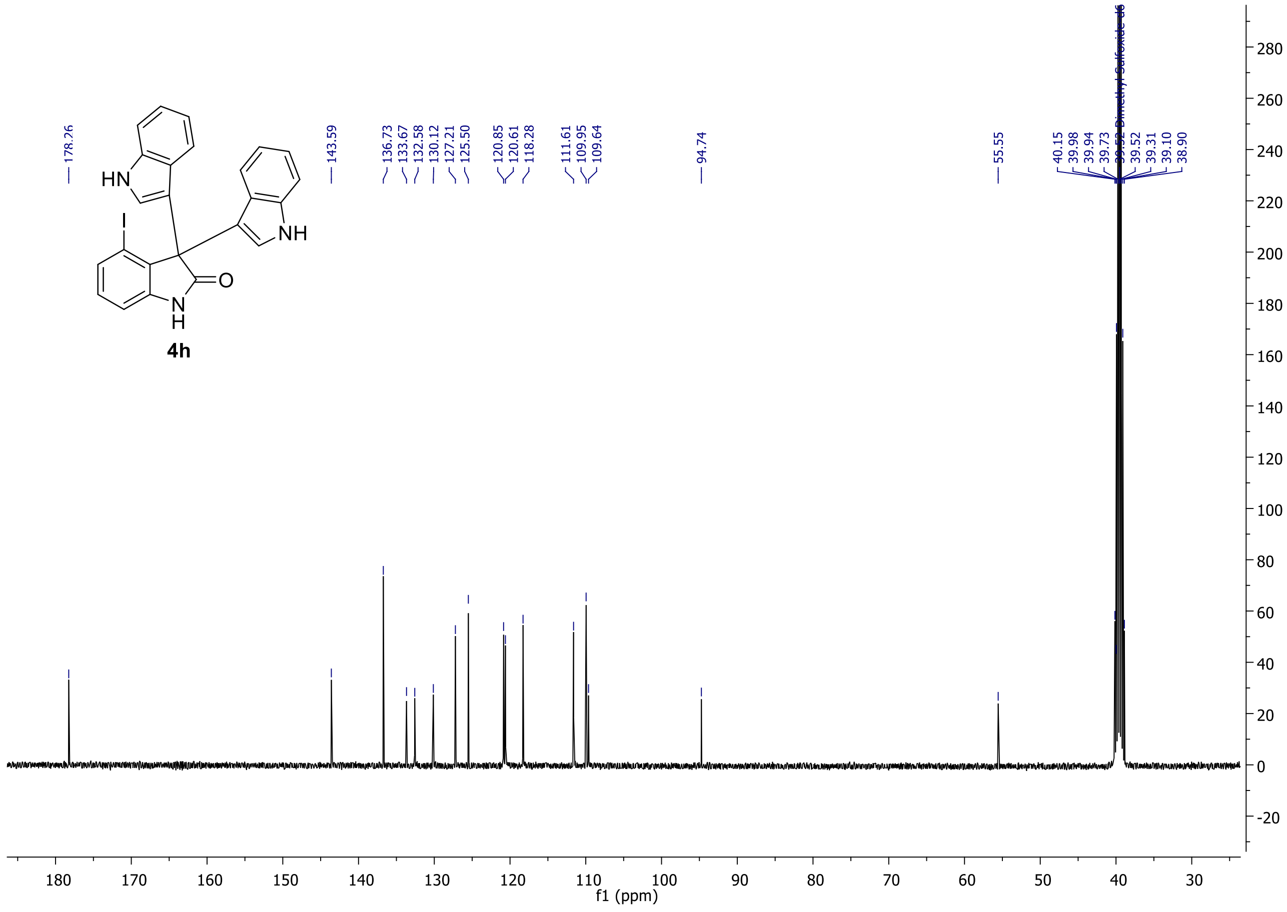
178.26
143.59
136.73
133.67
132.58
130.12
127.21
125.50
120.85
120.61
118.28
111.61
109.95
109.64
94.74
55.55
40.15
39.98
39.94
39.73
39.52
39.31
39.10
38.90
HN
NH
I
O
N
H
4h
f1 (ppm)

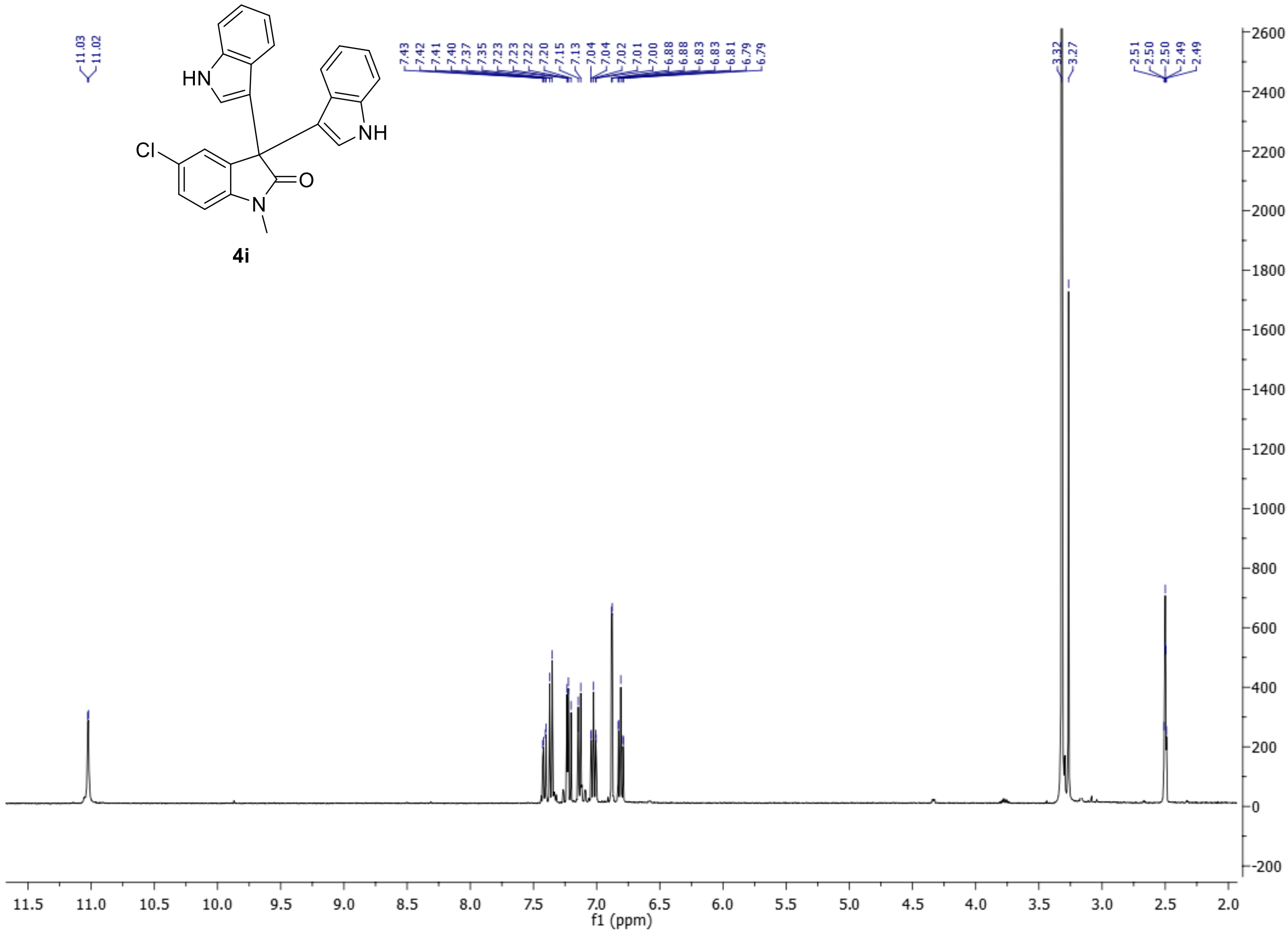
11.03
11.02
7.43
7.42
7.41
7.40
7.37
7.35
7.23
7.23
7.22
7.20
7.15
7.13
7.04
7.04
7.02
7.01
7.00
6.88
6.88
6.83
6.83
6.81
6.79
6.79
3.32
3.27
2.51
2.50
2.50
2.49
2.49
HN
NH
Cl
O
N
4i
f1 (ppm)

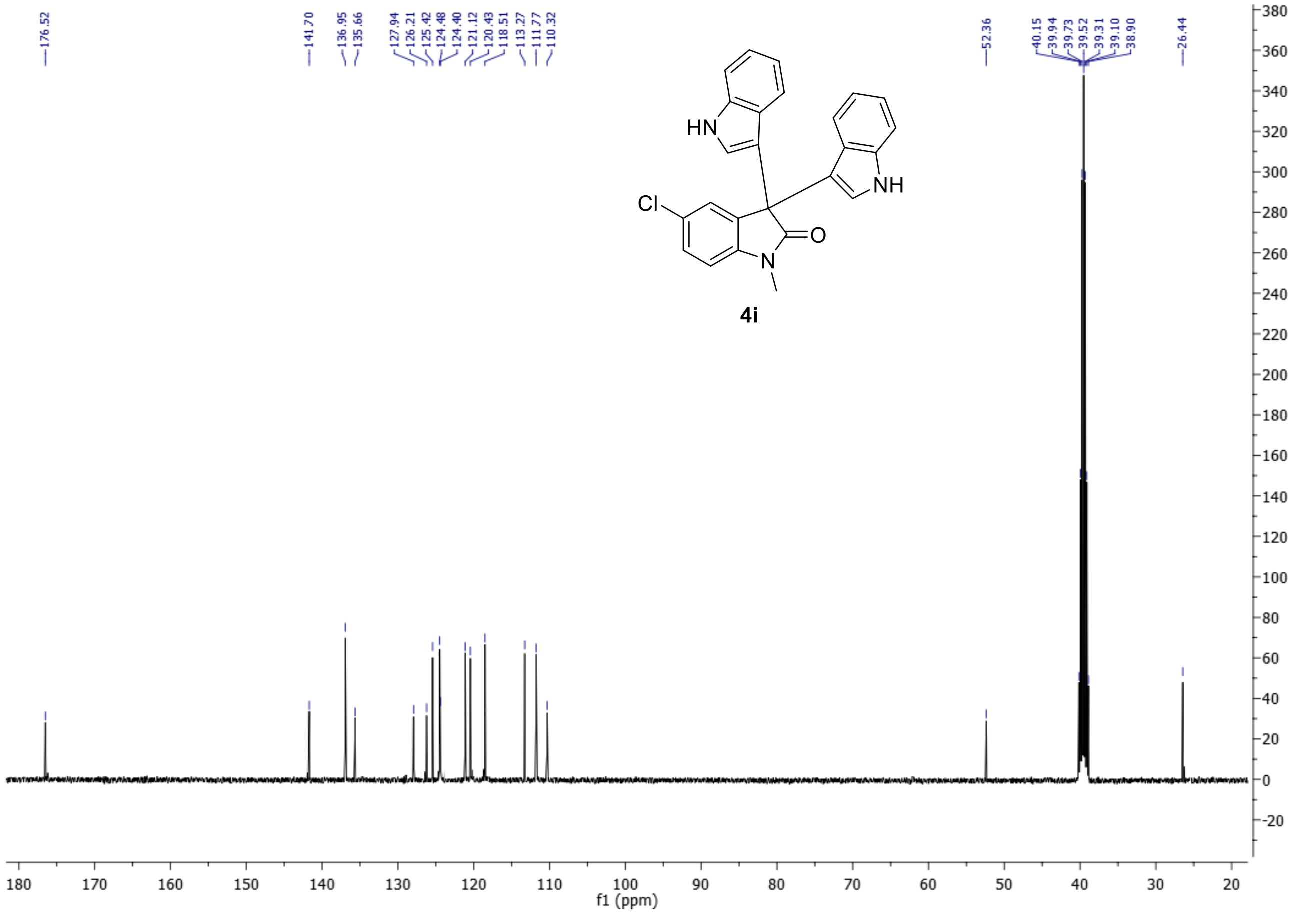
176.52
141.70
136.95
135.66
127.94
126.21
125.42
124.48
124.40
121.12
120.43
118.51
113.27
111.77
110.32
52.36
40.15
39.94
39.73
39.52
39.31
39.10
38.90
26.44
HN
NH
Cl
O
N
4i
f1 (ppm)

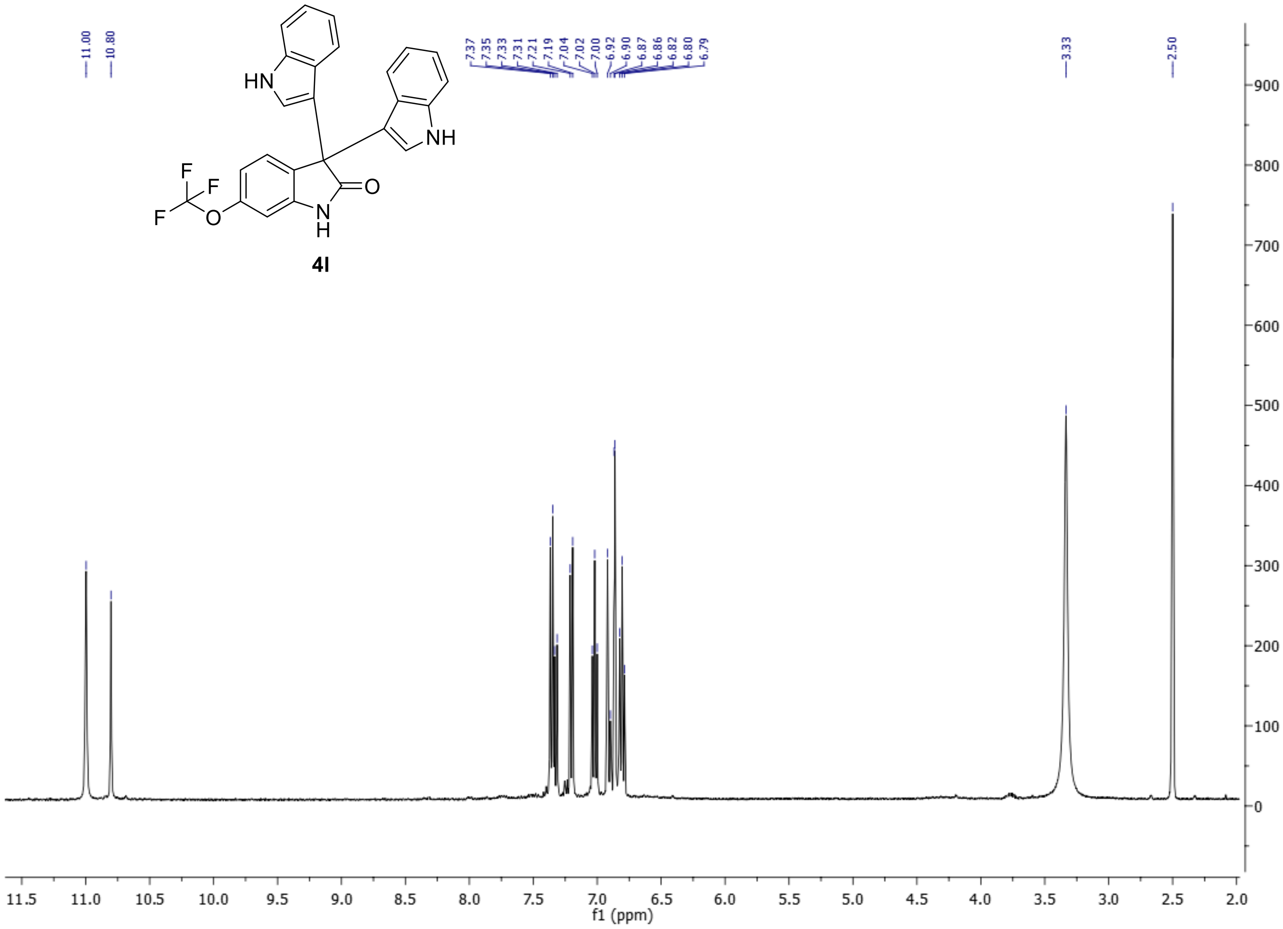
11.00
10.80
7.37
7.35
7.33
7.31
7.21
7.19
7.04
7.02
7.00
6.92
6.90
6.87
6.86
6.82
6.80
6.79
3.33
2.50
HN
NH
F
F
F
O
N
H
O
4l
f1 (ppm)

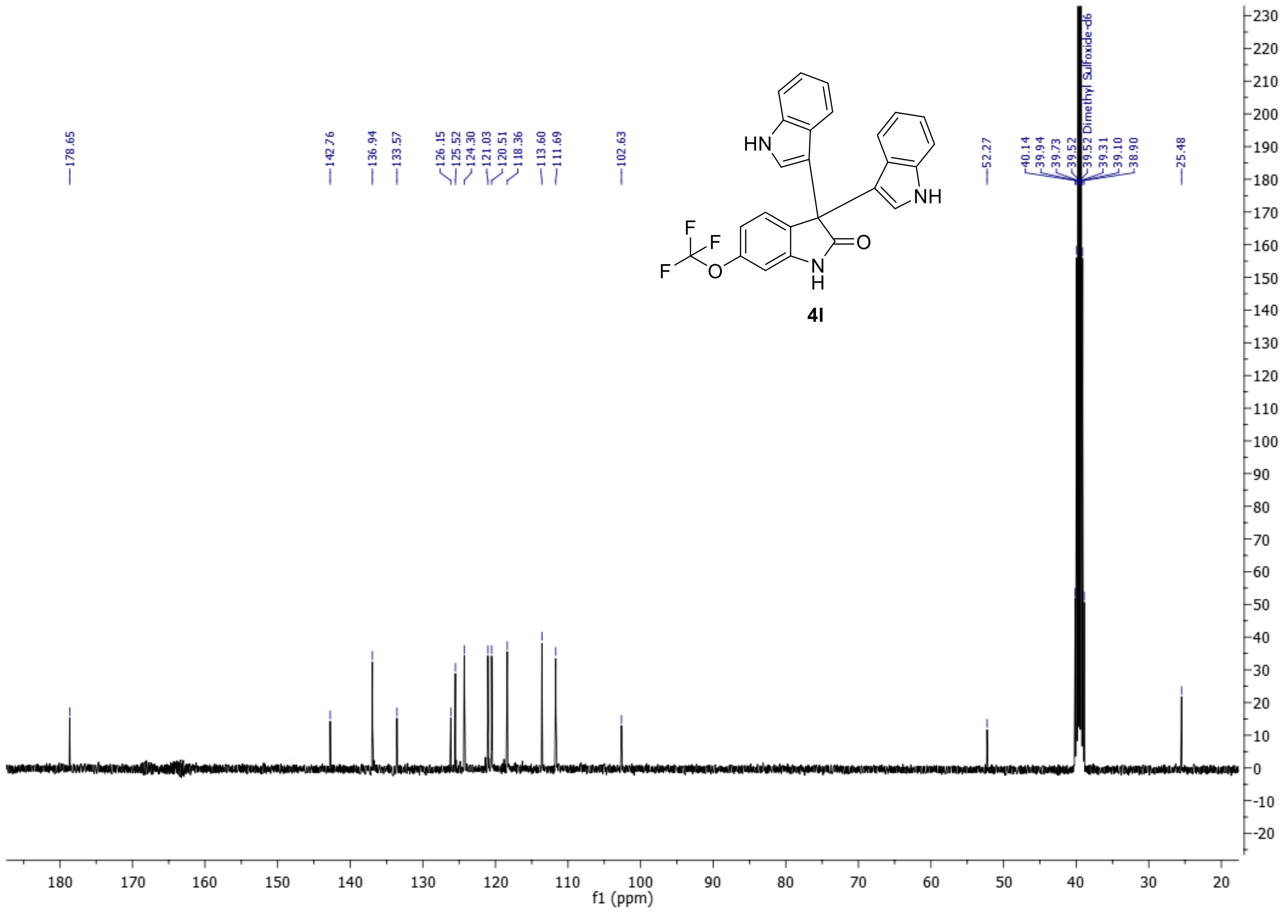
178.65
142.76
136.94
133.57
126.15
125.52
124.30
121.03
120.51
118.36
113.60
111.69
102.63
52.27
40.14
39.94
39.73
39.52
39.52 Dimethyl Sulfoxide-d6
39.31
39.10
38.90
25.48
HN
NH
F
F
F
O
N
H
O
4l
f1 (ppm)

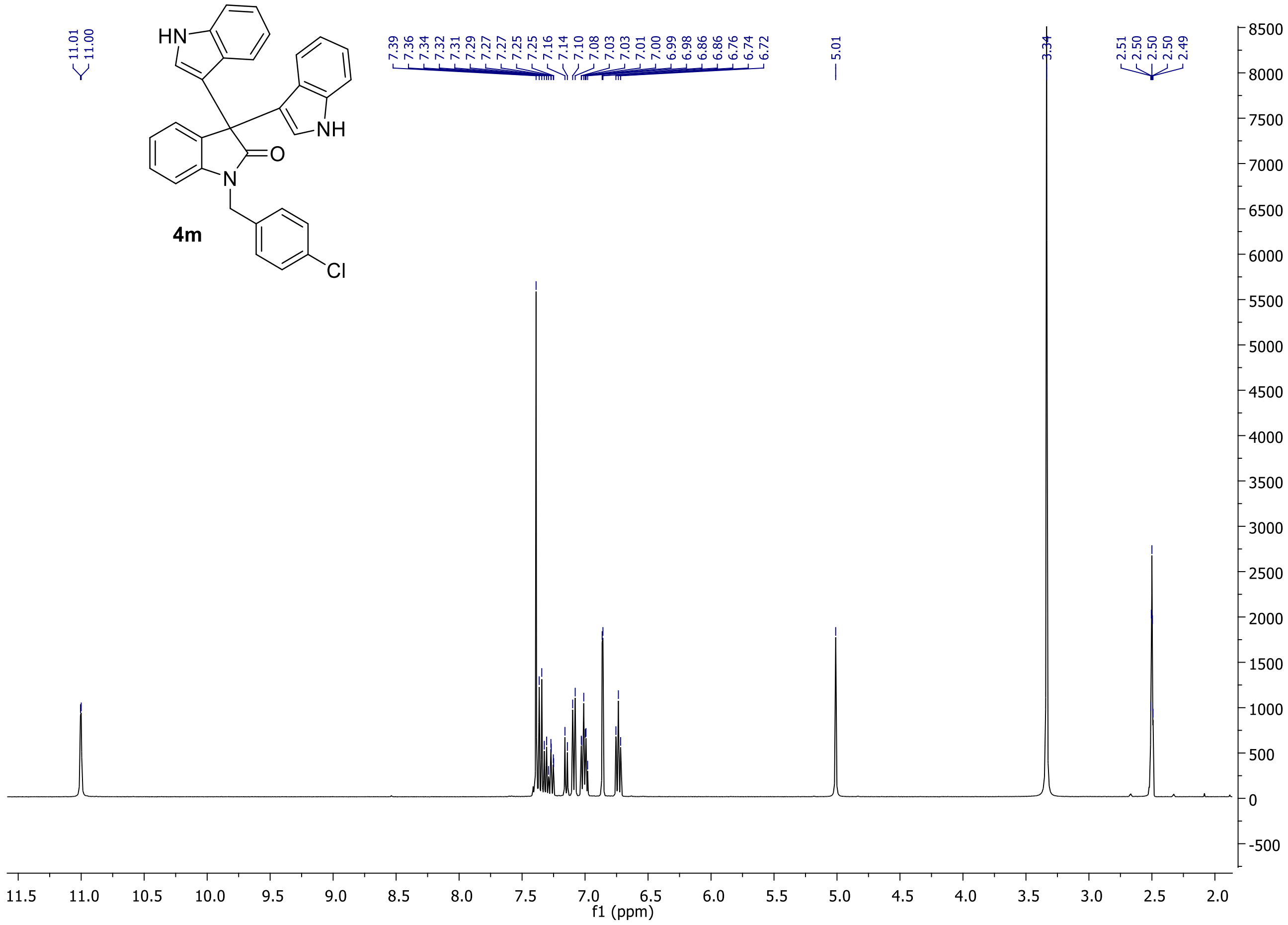

11.01
11.00
7.39
7.36
7.34
7.32
7.31
7.29
7.27
7.25
7.25
7.16
7.14
7.10
7.08
7.03
7.03
7.01
7.00
6.99
6.98
6.86
6.86
6.76
6.74
6.72
5.01
3.34
2.51
2.50
2.50
2.50
2.49
HN
NH
O
N
4m
Cl
f1 (ppm)

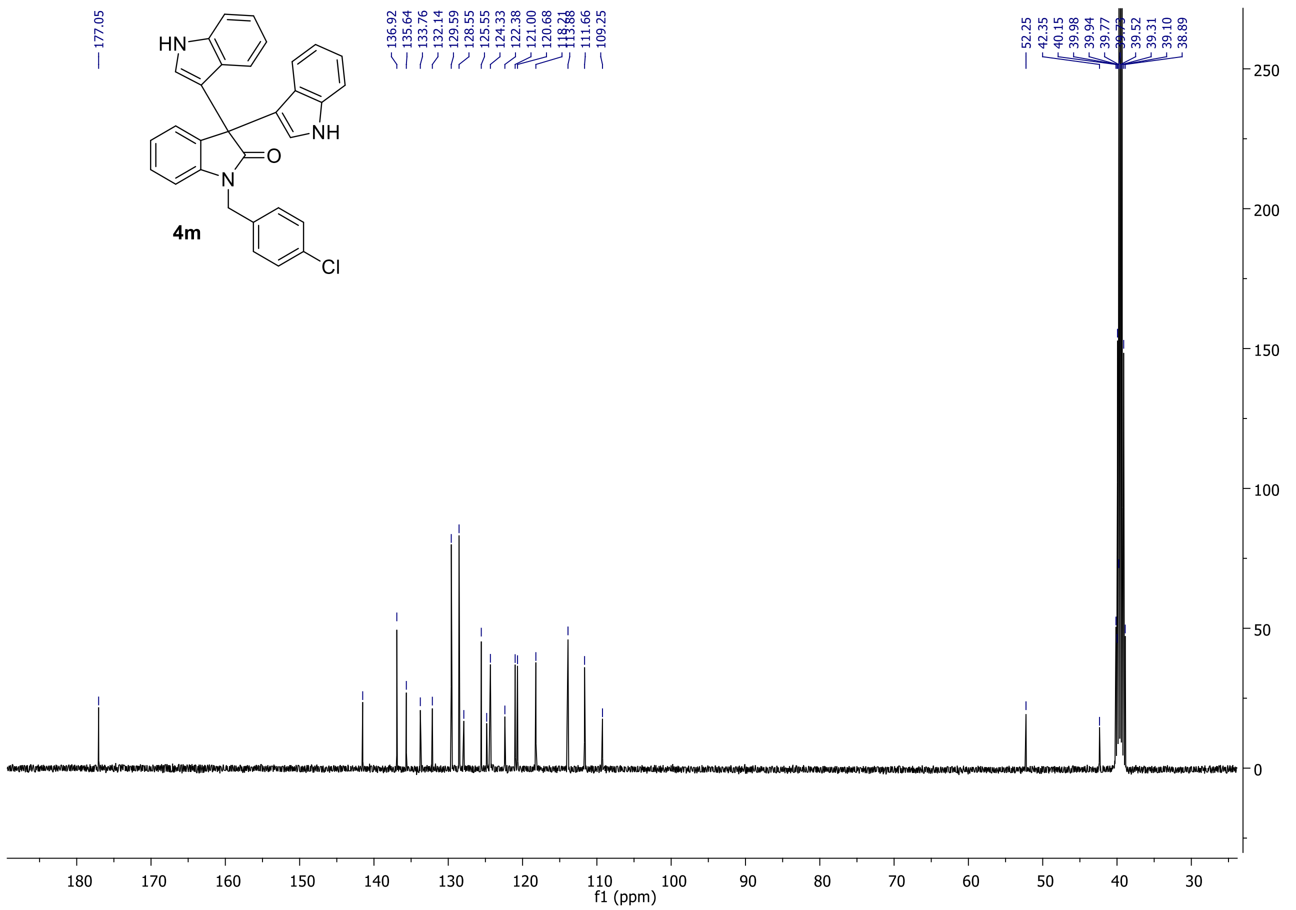

177.05
136.92
135.64
133.76
132.14
129.59
128.55
125.55
124.33
122.38
121.00
120.68
118.21
113.88
111.66
109.25
52.25
42.35
40.15
39.98
39.94
39.77
39.52
39.31
39.10
38.89
HN
NH
O
N
4m
Cl
f1 (ppm)

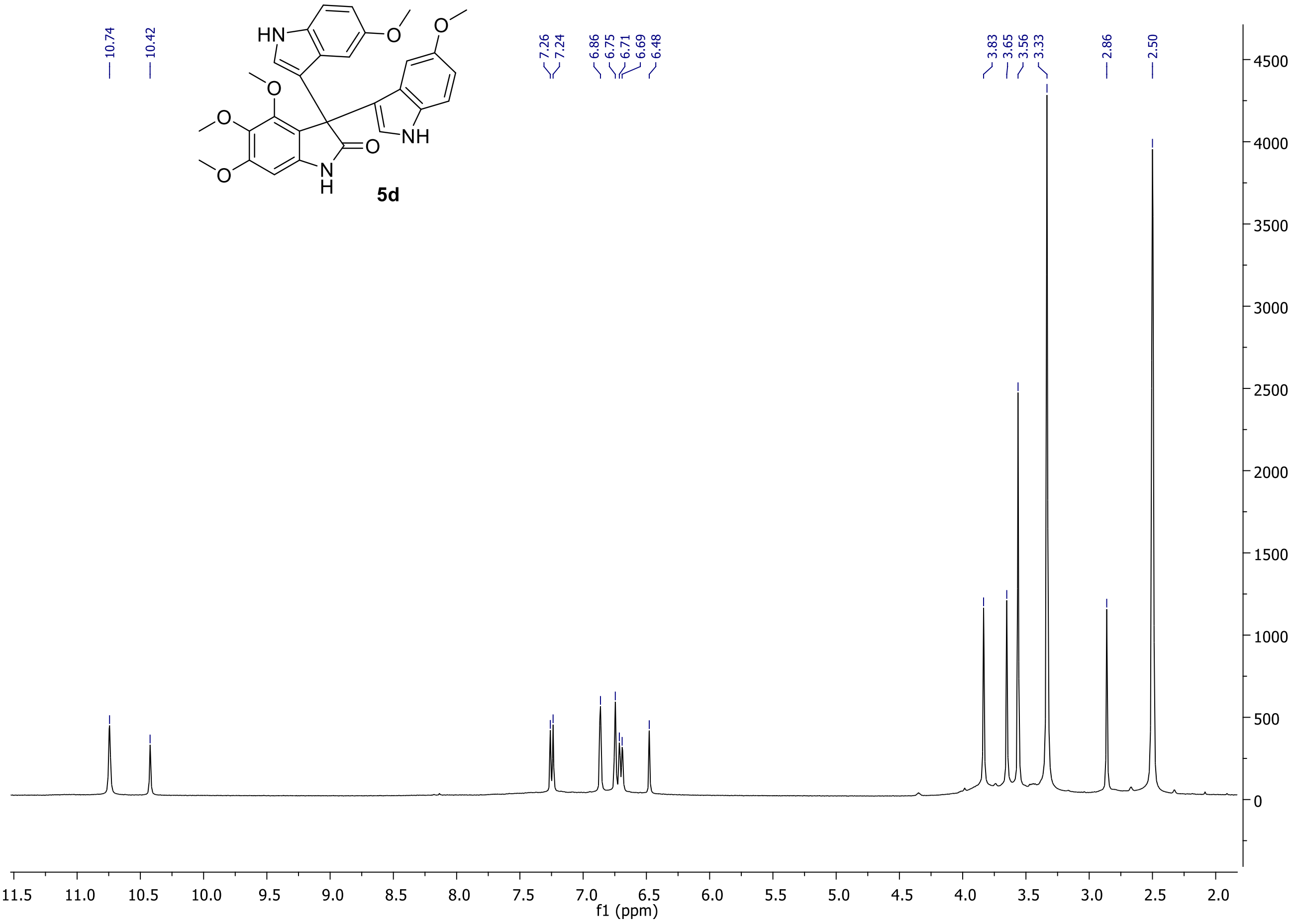
10.74
10.42
7.26
7.24
6.86
6.75
6.71
6.69
6.48
3.83
3.65
3.56
3.33
2.86
2.50
HN
NH
O
N
H
5d
f1 (ppm)

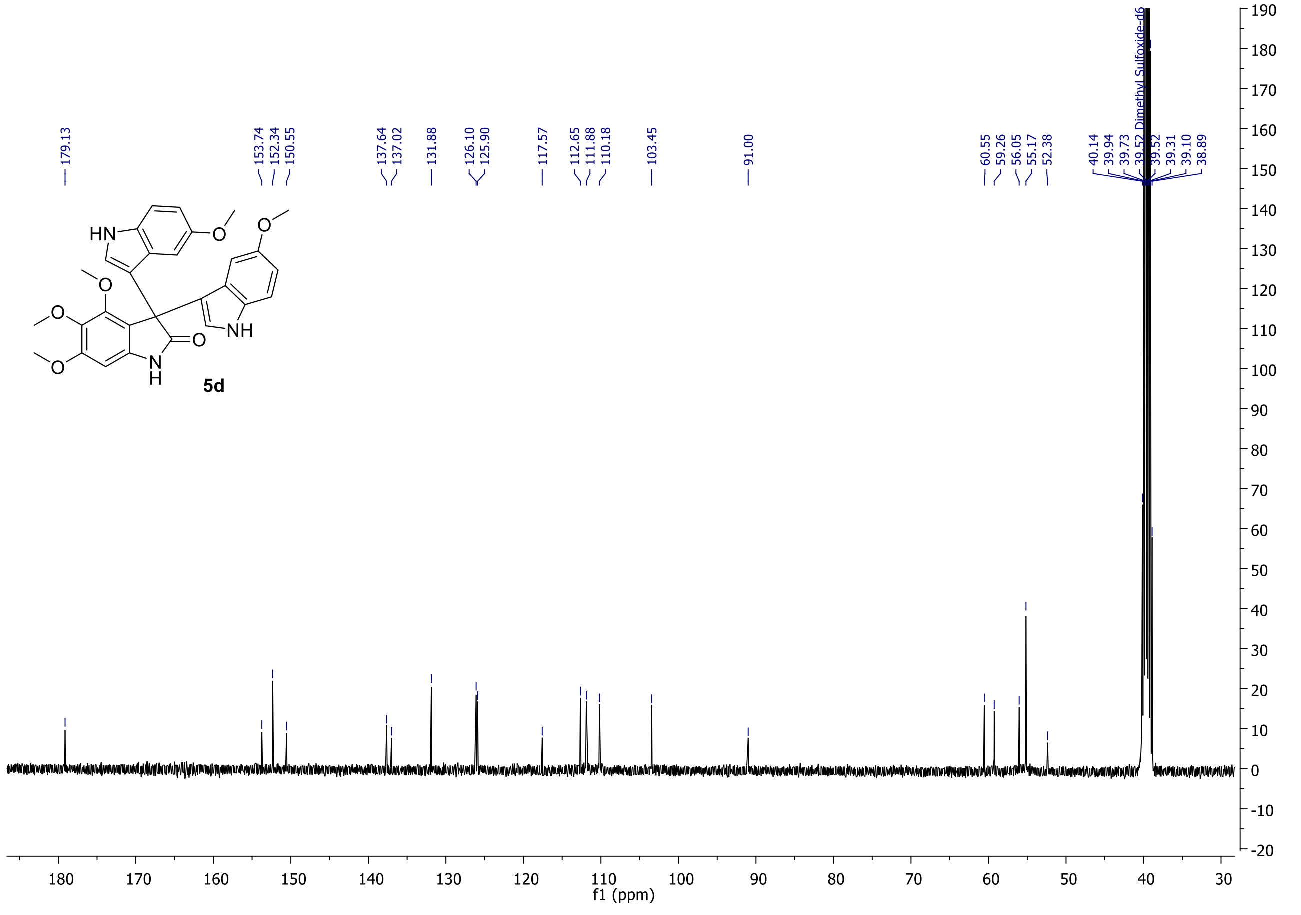
179.13
153.74
152.34
150.55
137.64
137.02
131.88
126.10
125.90
117.57
112.65
111.88
110.18
103.45
91.00
60.55
59.26
56.05
55.17
52.38
40.14
39.94
39.73
39.52 Dimethyl Sulfoxide-d6
39.52
39.31
39.10
38.89
HN
NH
O
N
H
5d
f1 (ppm)

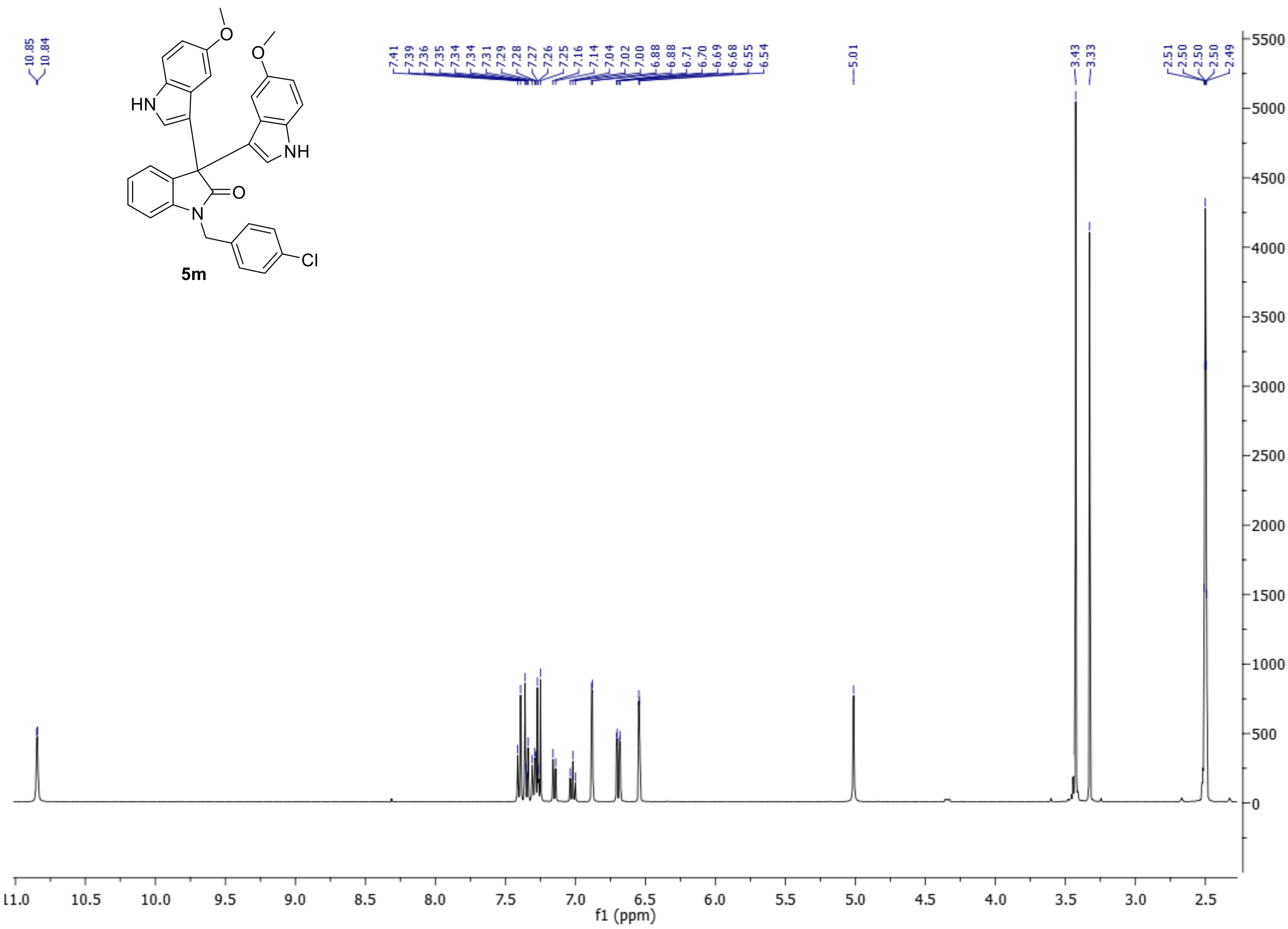

5m
f1 (ppm)

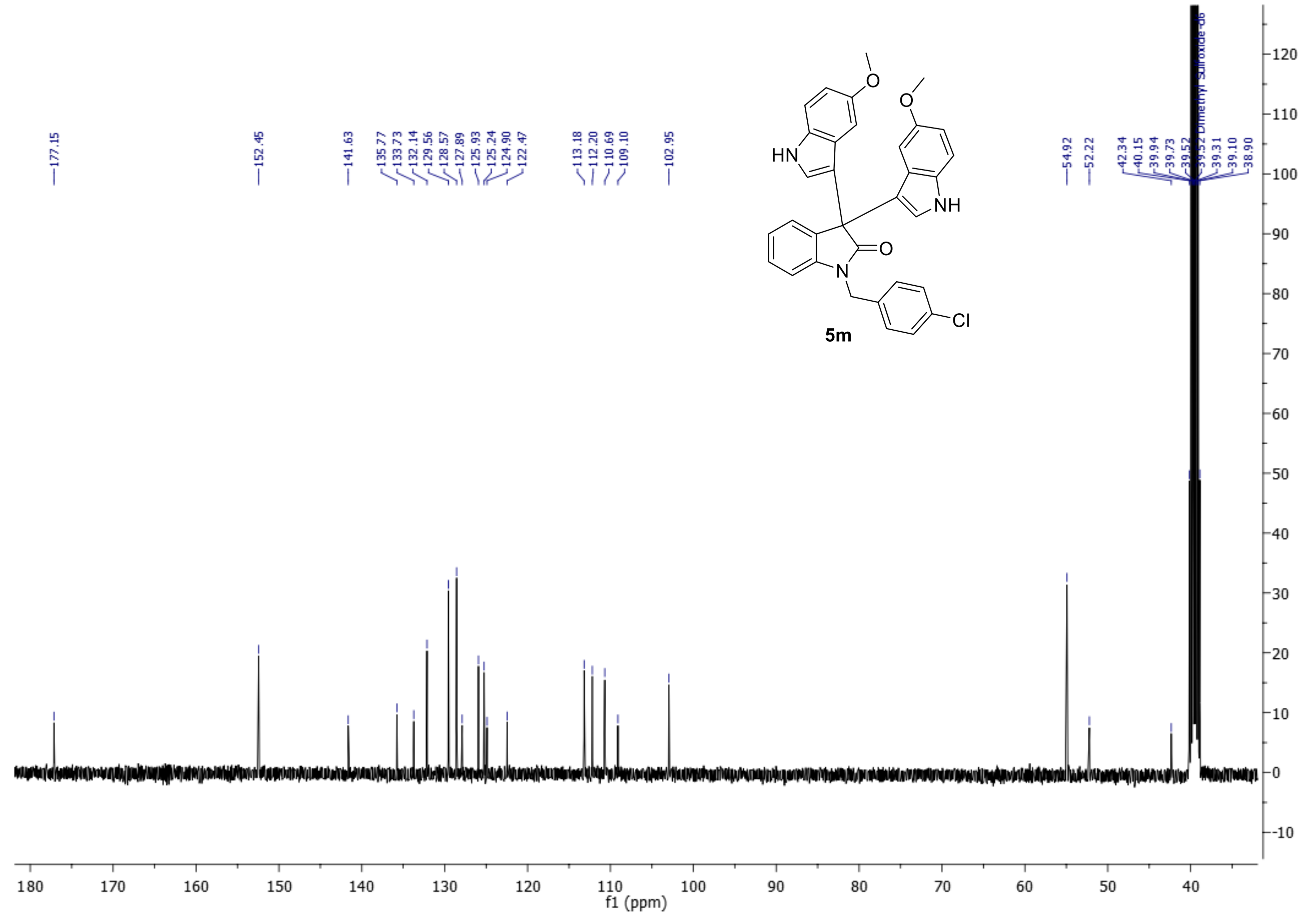

5m
f1 (ppm)

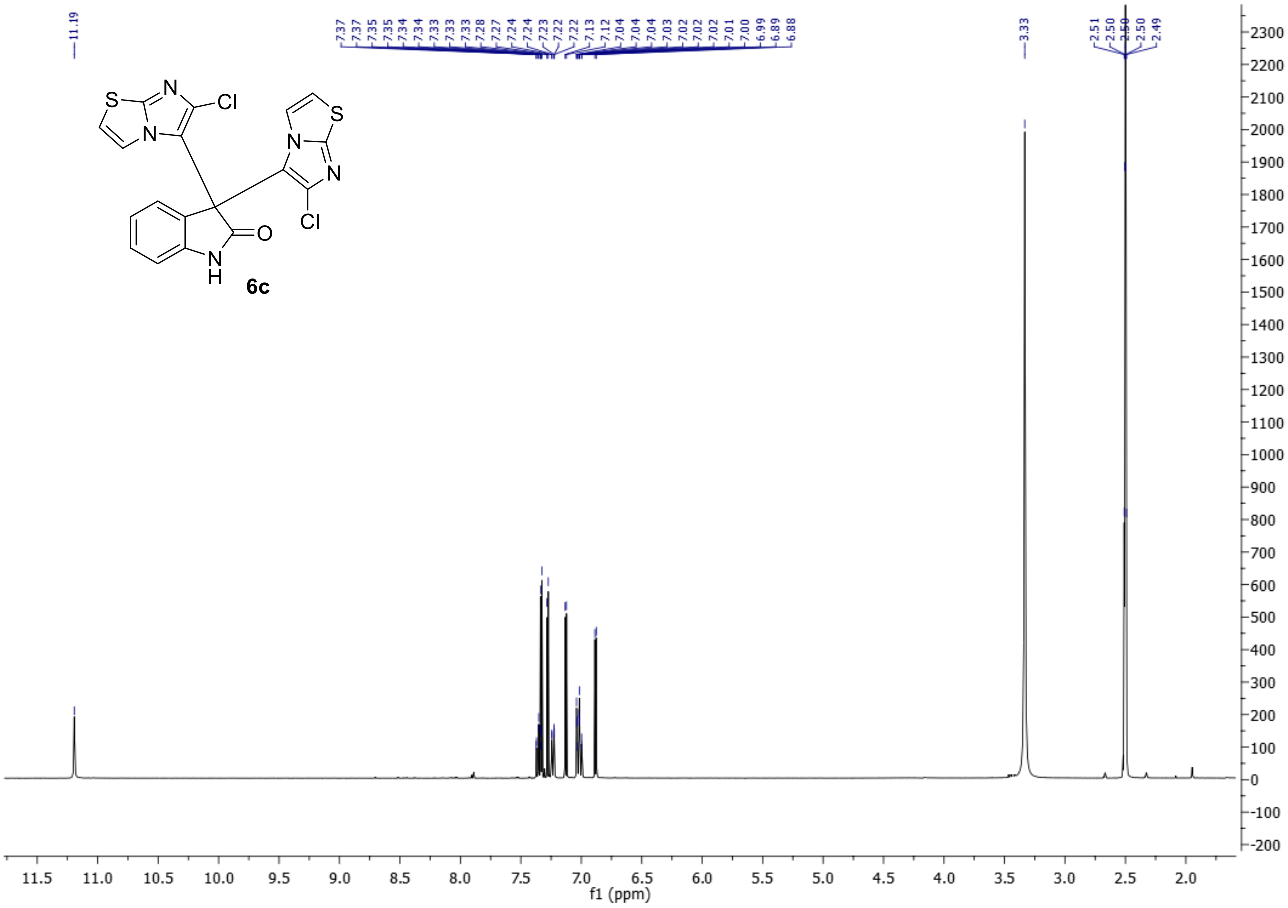
6c
f1 (ppm)

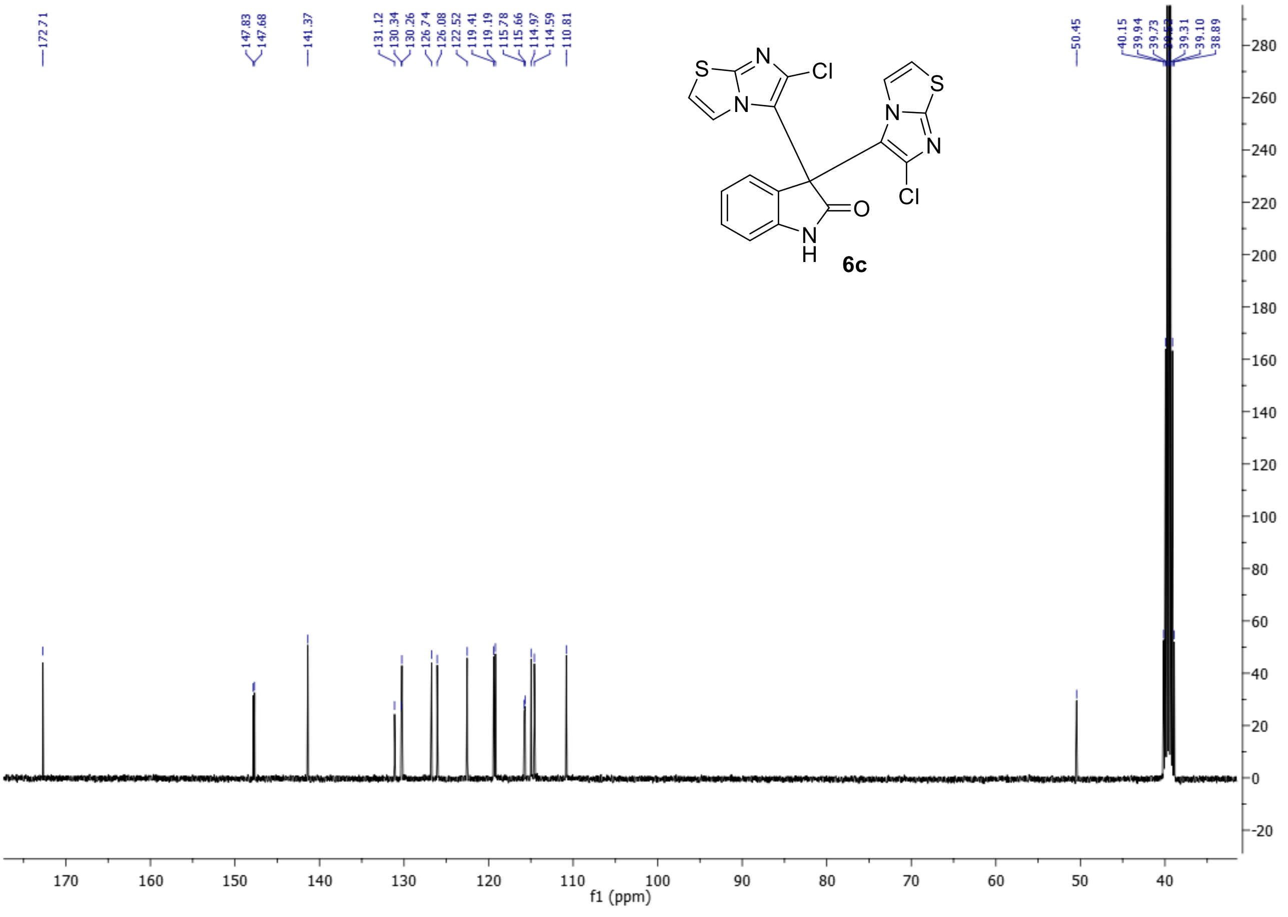
6c
f1 (ppm)

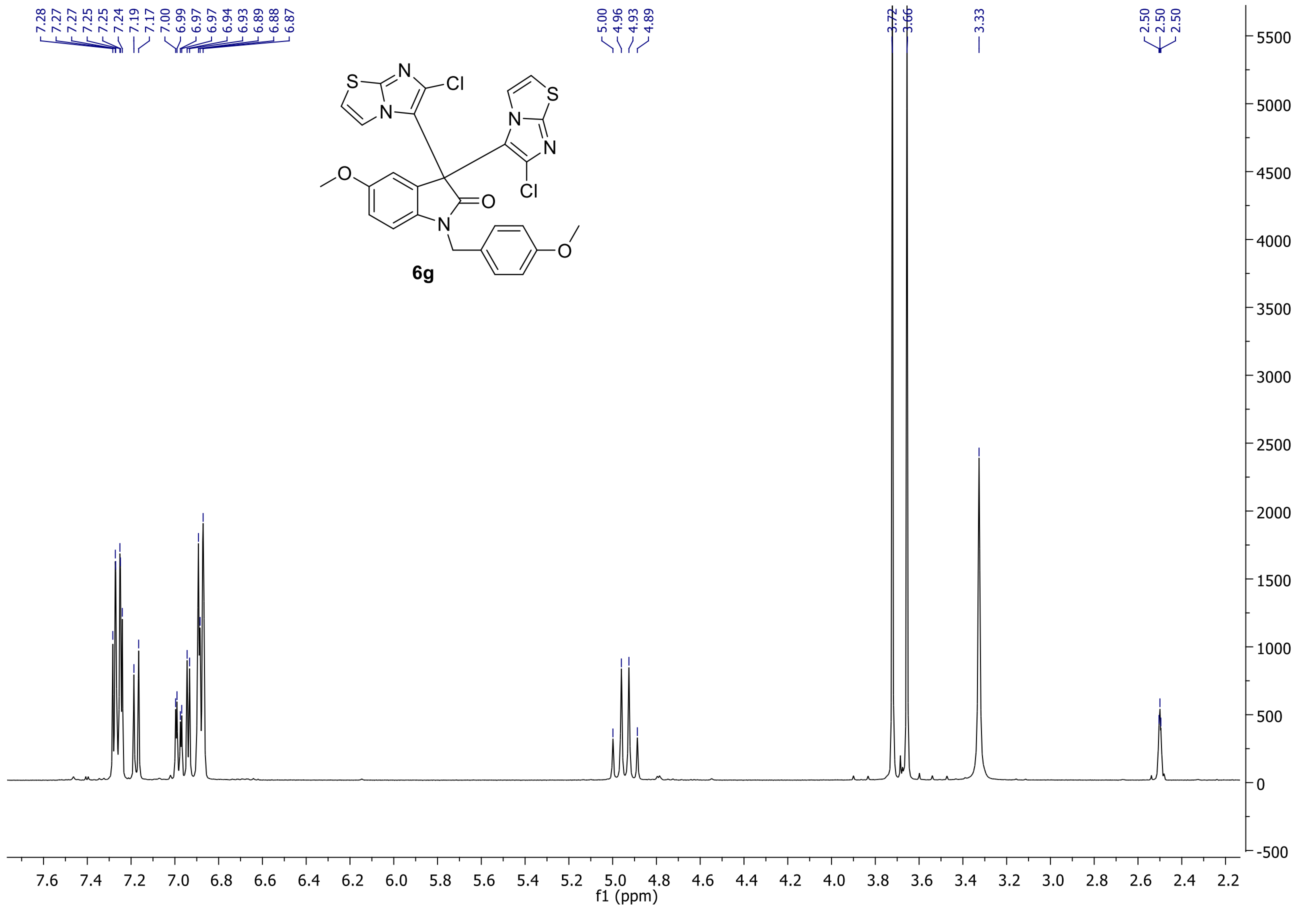
7.28 7.27 7.27 7.25 7.25 7.24 7.19 7.17 7.00 6.99 6.97 6.97 6.94 6.93 6.89 6.88 6.87
5.00 4.96 4.93 4.89
3.72 3.66
3.33
2.50 2.50 2.50
6g
f1 (ppm)

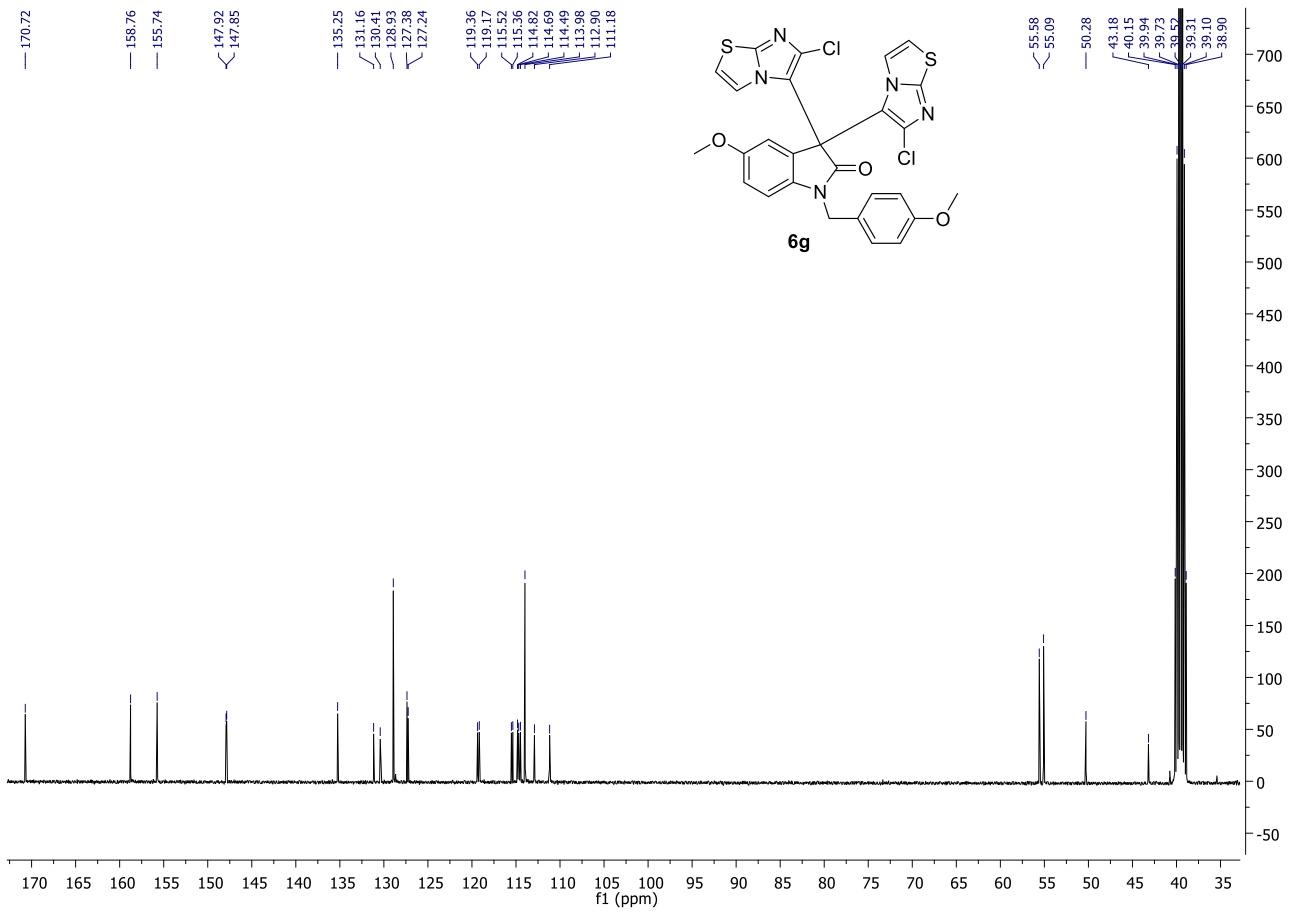
170.72
158.76
155.74
147.92 147.85
135.25
131.16 130.41 128.93 127.38 127.24
119.36 119.17 115.52 115.36 114.82 114.69 114.49 113.98 112.90 111.18
55.58 55.09
50.28
43.18 40.15 39.94 39.73 39.52 39.31 39.10 38.90
6g
f1 (ppm)

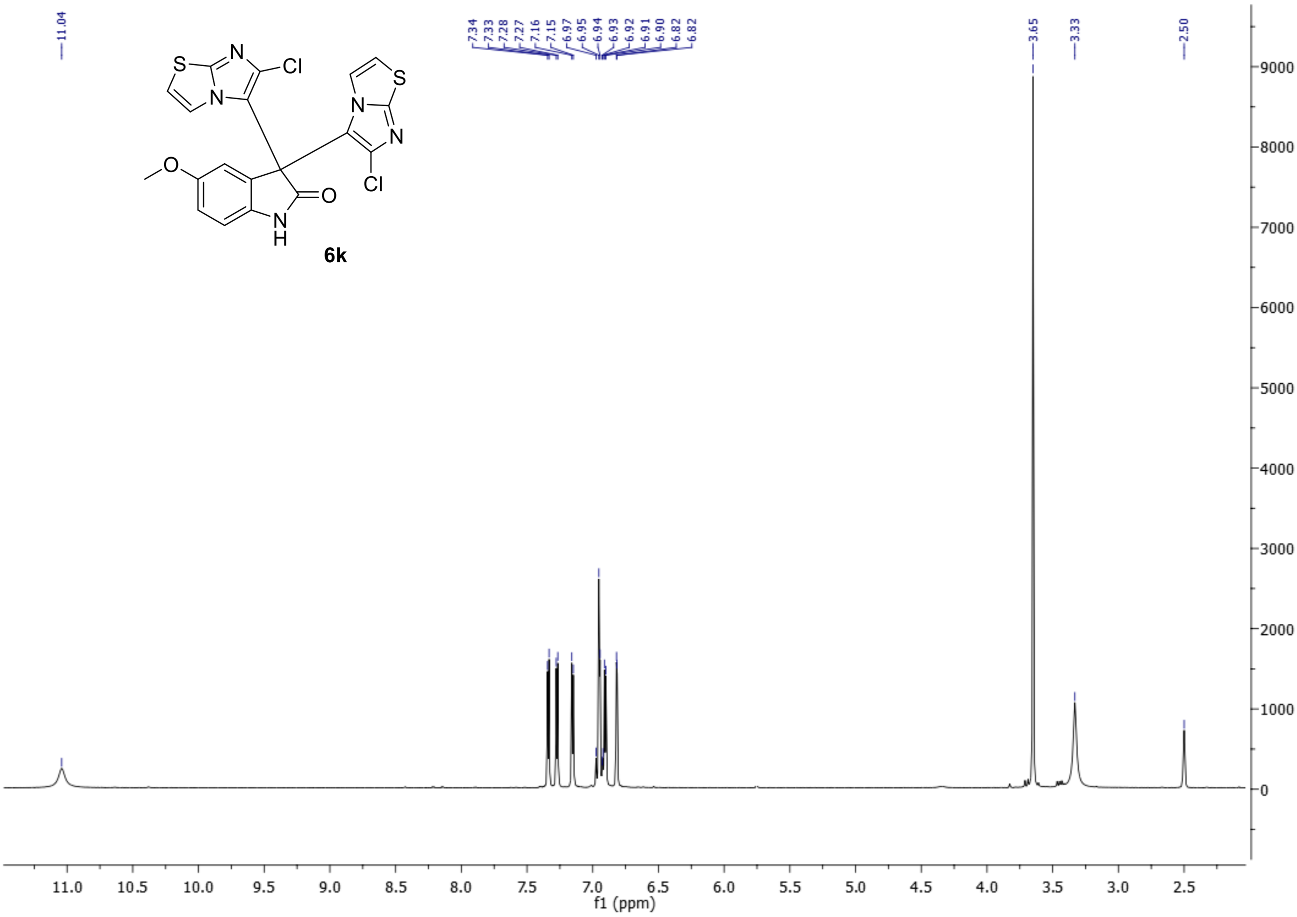
11.04
7.34
7.33
7.28
7.27
7.16
7.15
6.97
6.95
6.94
6.93
6.92
6.91
6.90
6.82
6.82
3.65
3.33
2.50
6k
f1 (ppm)

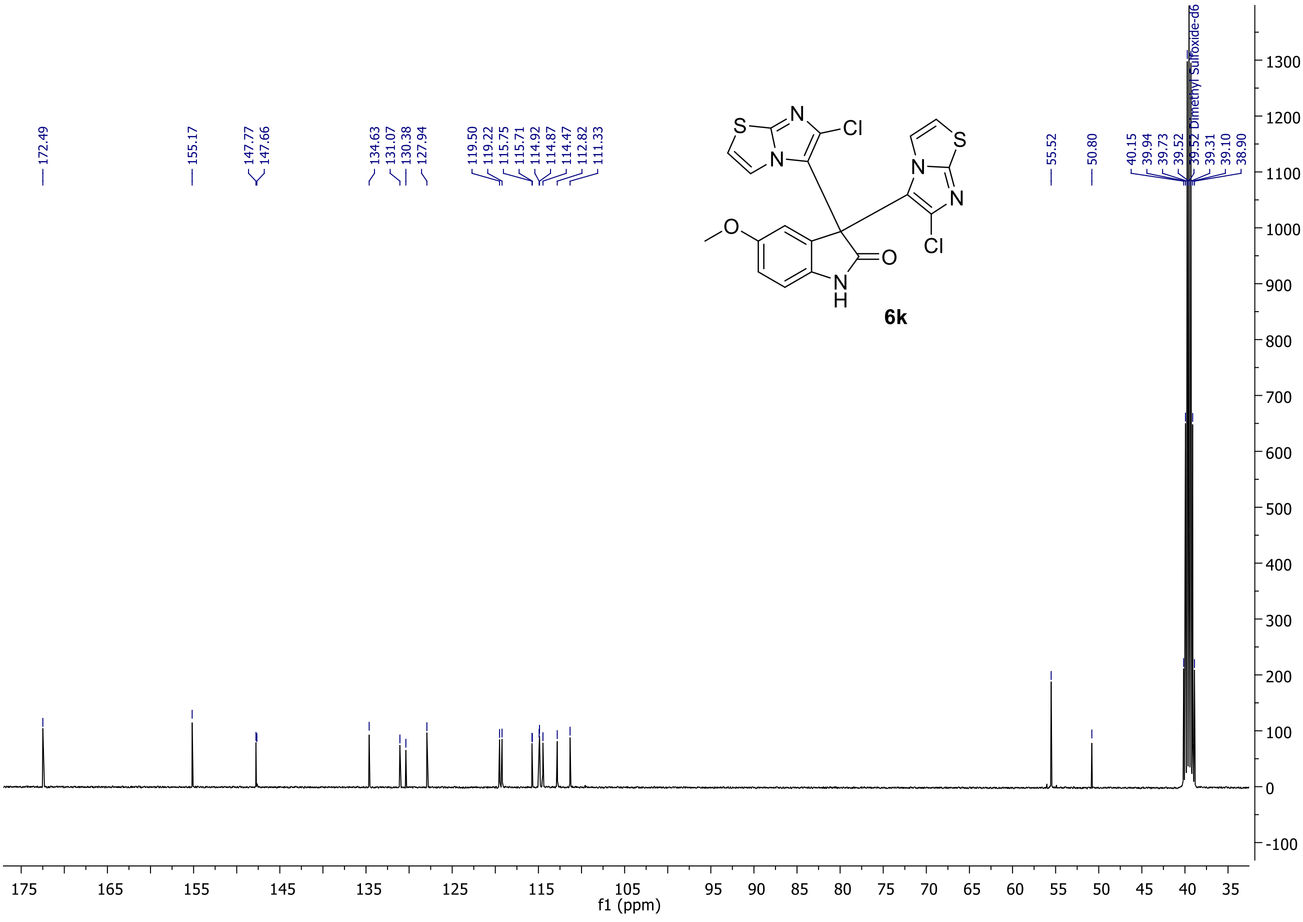
172.49
155.17
147.77
147.66
134.63
131.07
130.38
127.94
119.50
119.22
115.75
115.71
114.92
114.87
114.47
112.82
111.33
55.52
50.80
40.15
39.94
39.73
39.52
39.52 Dimethyl Sulfoxide-d6
39.31
39.10
38.90
6k
f1 (ppm)

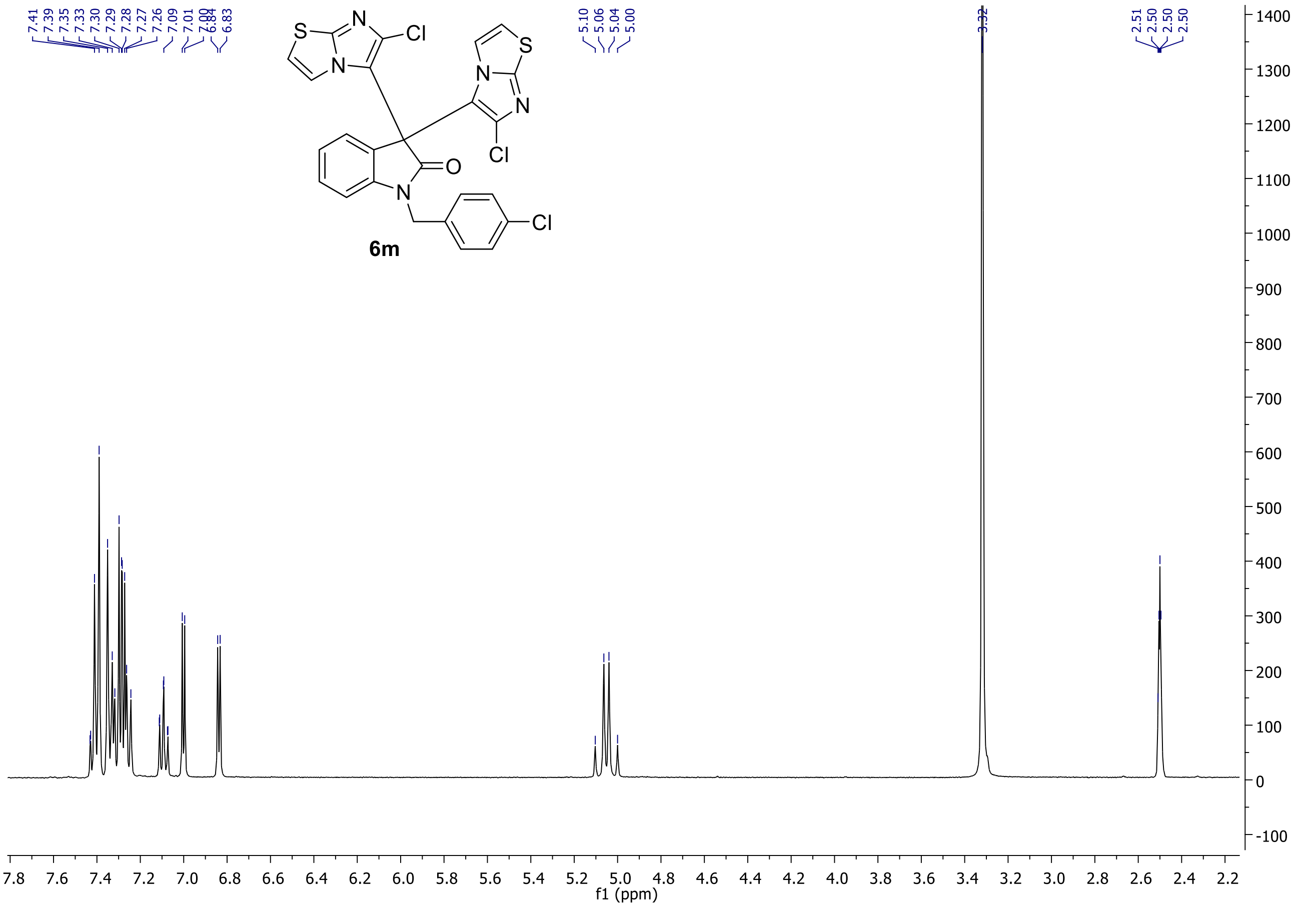
7.41
7.39
7.35
7.33
7.30
7.29
7.28
7.27
7.26
7.09
7.01
6.83
5.10
5.06
5.04
5.00
2.51
2.50
2.50
2.50
Cl
S
N
6m
f1 (ppm)

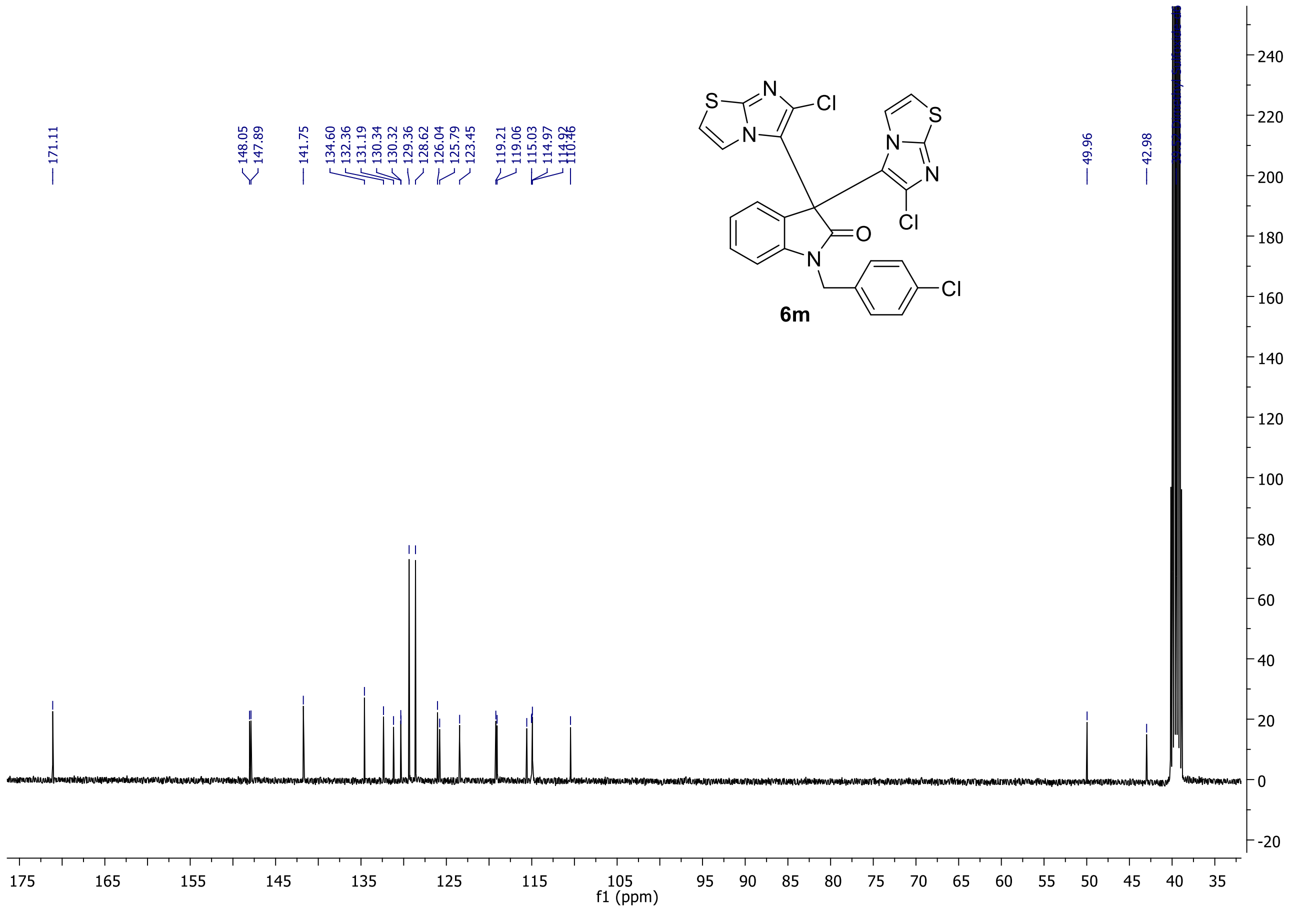
171.11
148.05
147.89
141.75
134.60
132.36
131.19
130.34
130.32
129.36
128.62
126.04
125.79
123.45
119.21
119.06
115.03
114.97
49.96
42.98
Cl
S
N
6m
f1 (ppm)

HRMS spectra of the described compounds

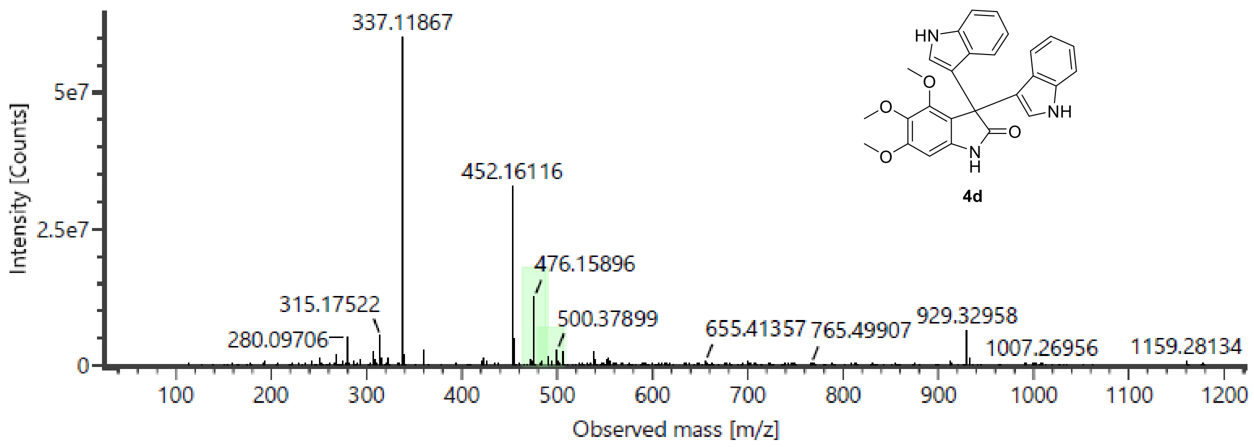

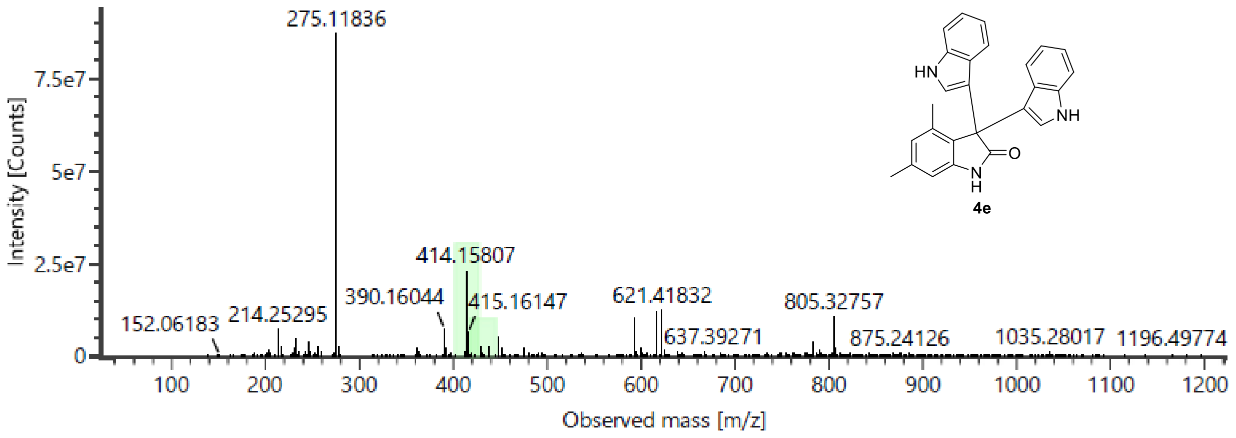

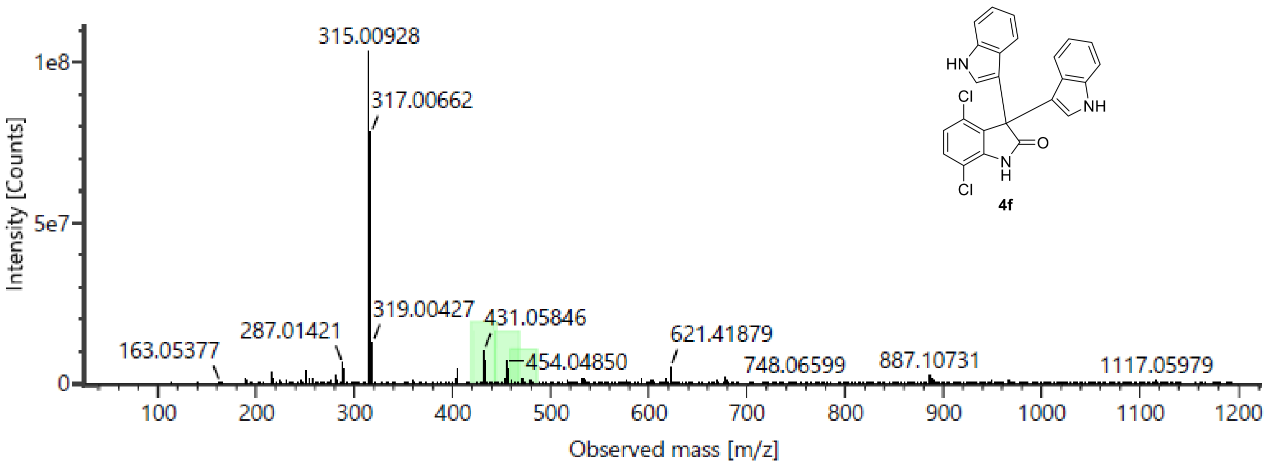

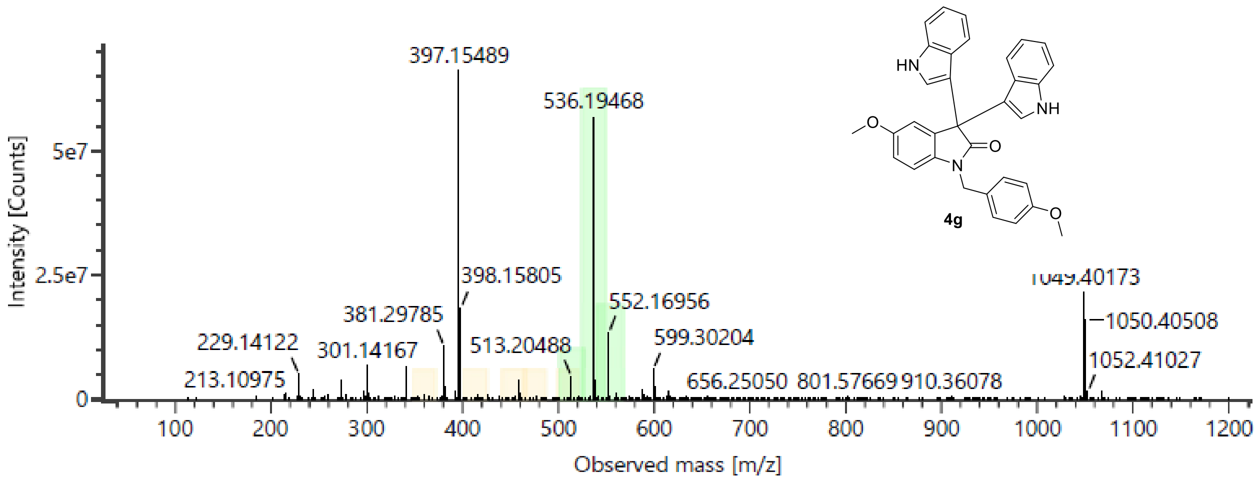
Intensity [Counts]
5e7
2.5e7
0
397.15489
536.19468
398.15805
381.29785
229.14122
301.14167
513.20488
552.16956
599.30204
213.10975
656.25050
801.57669
910.36078
1049.40173
1050.40508
1052.41027
100 200 300 400 500 600 700 800 900 1000 1100 1200
Observed mass [m/z]
4g

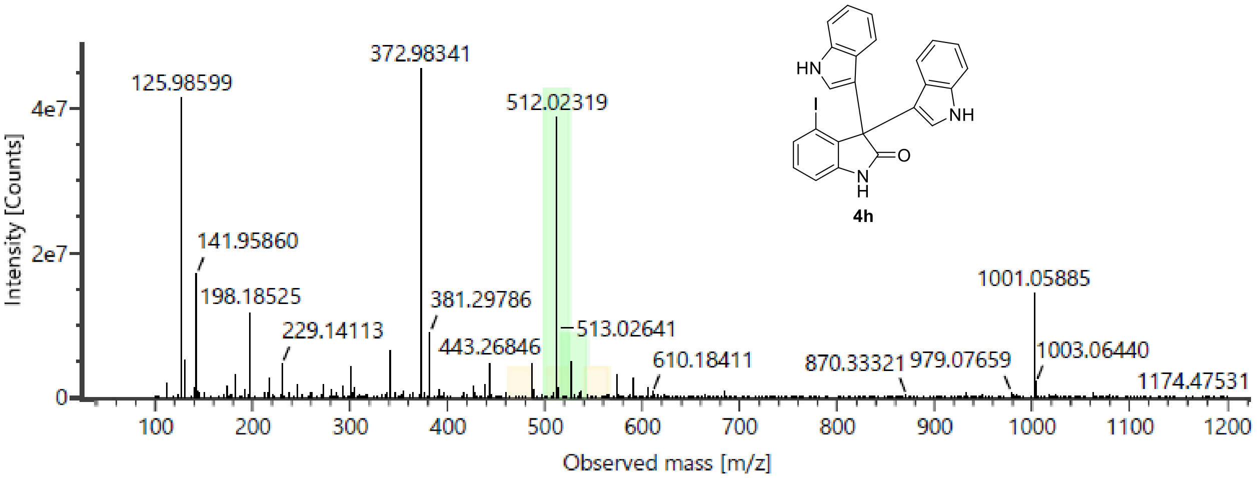
Intensity [Counts]
4e7
2e7
0
125.98599
372.98341
512.02319
141.95860
198.18525
229.14113
381.29786
443.26846
513.02641
610.18411
870.33321
979.07659
1001.05885
1003.06440
1174.47531
100 200 300 400 500 600 700 800 900 1000 1100 1200
Observed mass [m/z]
4h

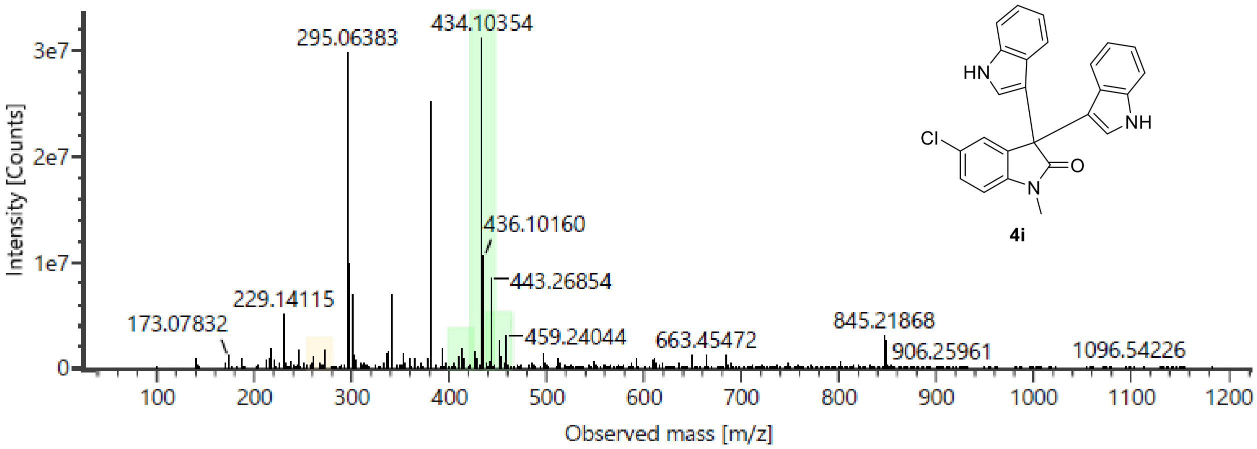
Intensity [Counts]
3e7
2e7
1e7
0
295.06383
434.10354
436.10160
443.26854
459.24044
229.14115
173.07832
663.45472
845.21868
906.25961
1096.54226
100 200 300 400 500 600 700 800 900 1000 1100 1200
Observed mass [m/z]
4i

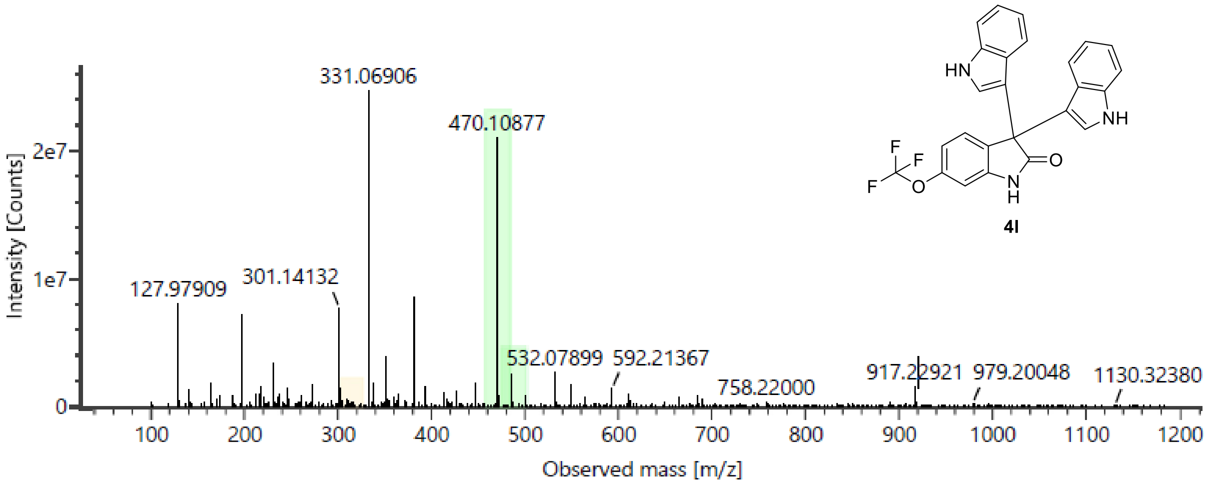

331.06906
470.10877
2e7
1e7
0
Intensity [Counts]
127.97909
301.14132
532.07899
592.21367
758.22000
917.22921
979.20048
1130.32380
100 200 300 400 500 600 700 800 900 1000 1100 1200
Observed mass [m/z]
HN
NH
F
F
F
O
O
N
H
4l

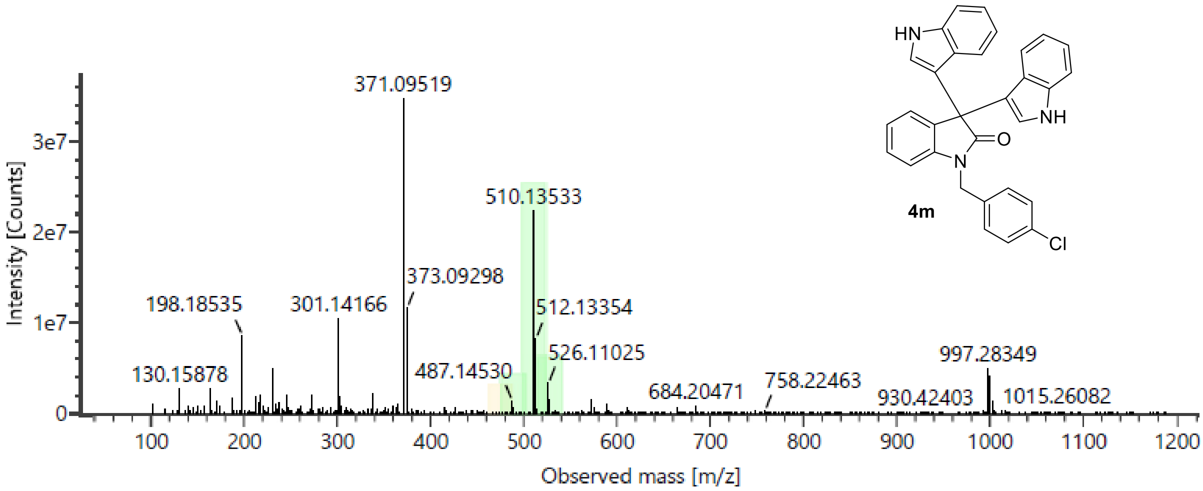

371.09519
510.13533
373.09298
198.18535
301.14166
512.13354
526.11025
130.15878
487.14530
684.20471
758.22463
930.42403
997.28349
1015.26082
3e7
2e7
1e7
0
Intensity [Counts]
100 200 300 400 500 600 700 800 900 1000 1100 1200
Observed mass [m/z]
HN
NH
N
O
Cl
4m

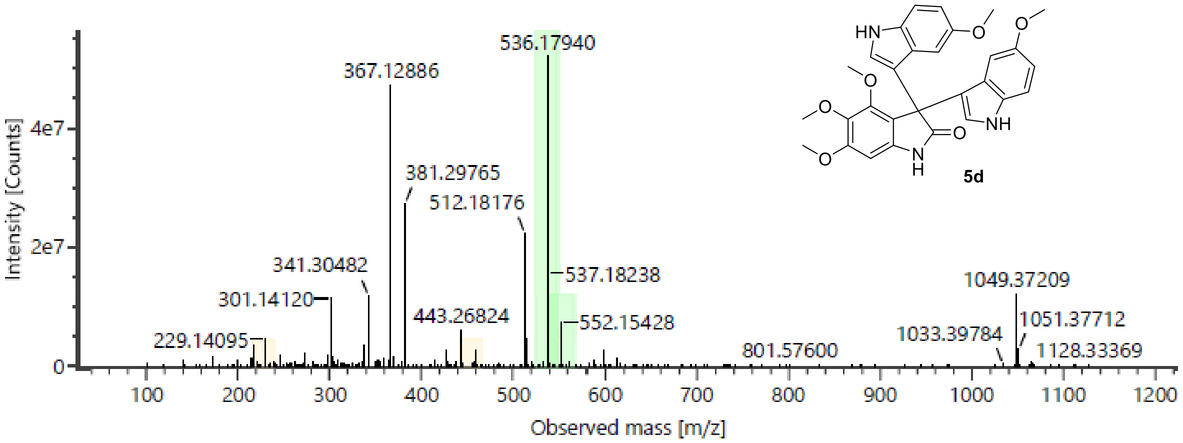

536.17940
367.12886
381.29765
512.18176
341.30482
301.14120
537.18238
443.26824
552.15428
229.14095
801.57600
1049.37209
1051.37712
1033.39784
1128.33369
4e7
2e7
0
Intensity [Counts]
100 200 300 400 500 600 700 800 900 1000 1100 1200
Observed mass [m/z]
HN
NH
O
N
H
5d

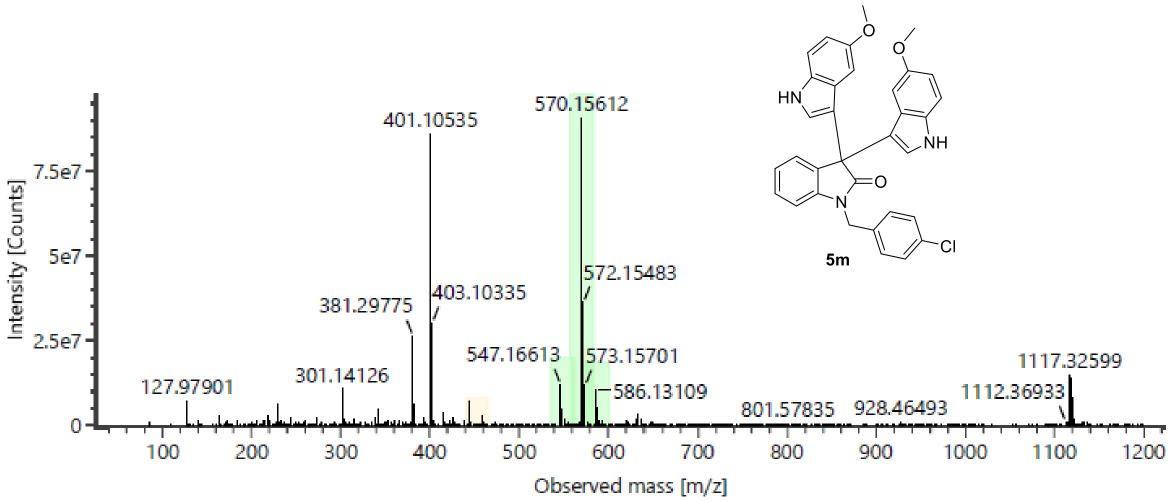
5m
Intensity [Counts]
Observed mass [m/z]
570.15612
401.10535
572.15483
403.10335
381.29775
547.16613
573.15701
586.13109
301.14126
127.97901
801.57835
928.46493
1117.32599
1112.36933

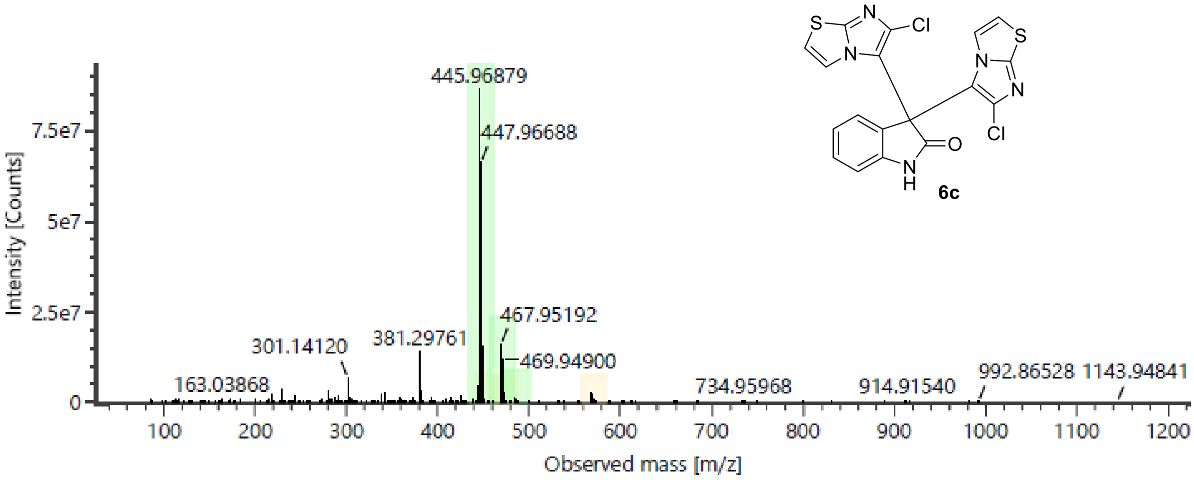
6c
Intensity [Counts]
Observed mass [m/z]
445.96879
447.96688
467.95192
469.94900
381.29761
301.14120
163.03868
734.95968
914.91540
992.86528
1143.94841

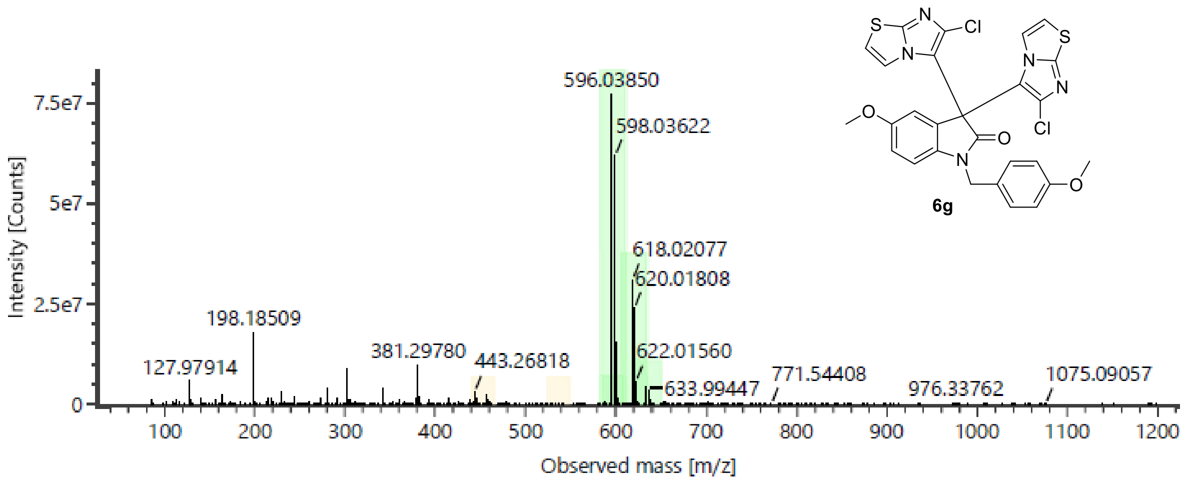
6g
Intensity [Counts]
Observed mass [m/z]
596.03850
598.03622
618.02077
620.01808
622.01560
633.99447
198.18509
381.29780
443.26818
127.97914
771.54408
976.33762
1075.09057

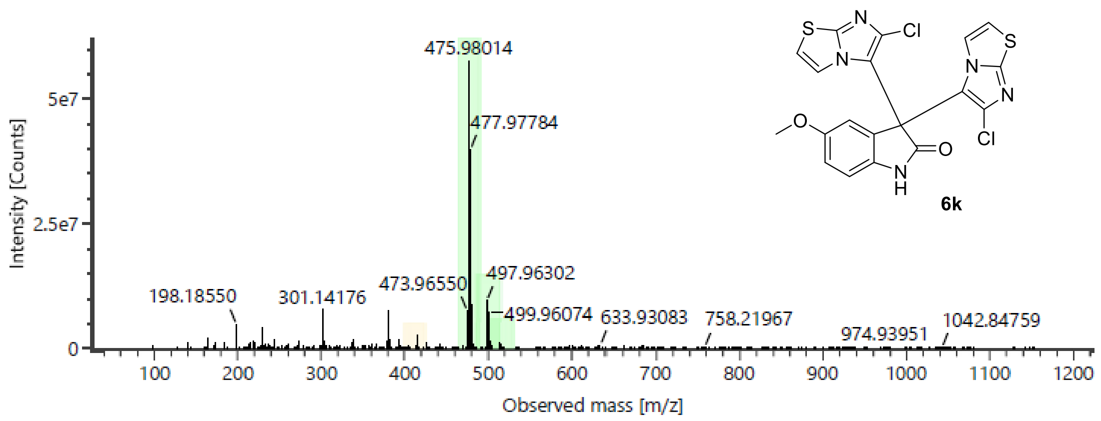
6k
Intensity [Counts]
Observed mass [m/z]
5e7
2.5e7
0
100
200
300
400
500
600
700
800
900
1000
1100
1200
475.98014
477.97784
473.96550
497.96302
499.96074
198.18550
301.14176
633.93083
758.21967
974.93951
1042.84759

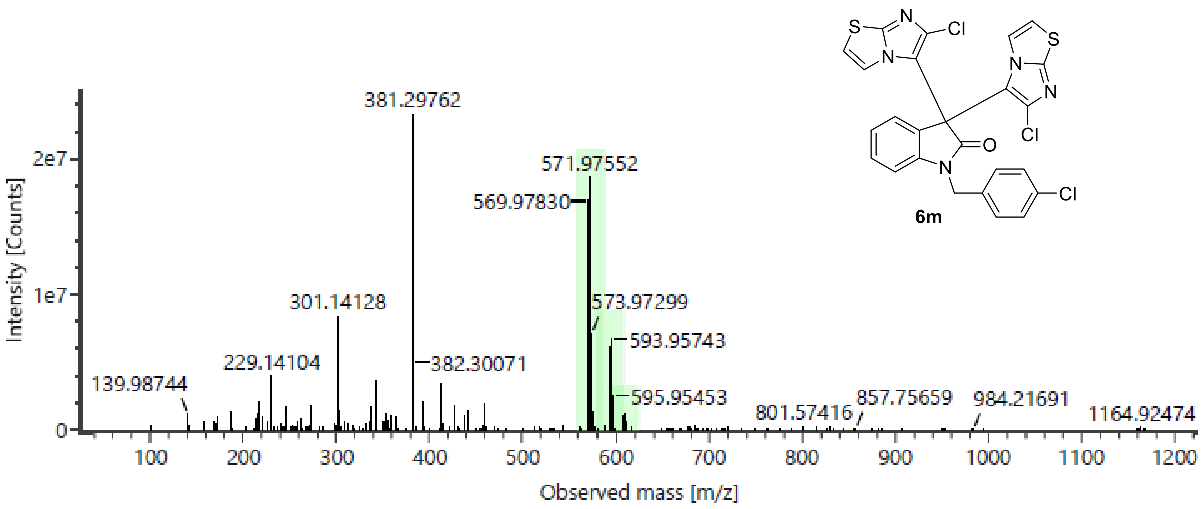
6m
Intensity [Counts]
Observed mass [m/z]
2e7
1e7
0
100
200
300
400
500
600
700
800
900
1000
1100
1200
381.29762
571.97552
569.97830
573.97299
593.95743
595.95453
301.14128
229.14104
382.30071
139.98744
801.57416
857.75659
984.21691
1164.92474

Figure S1

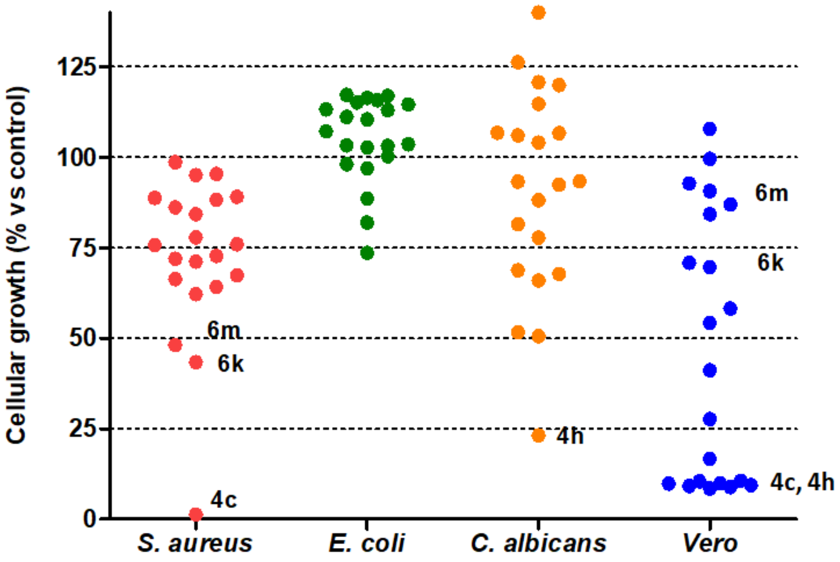

General overview on the effect of the 18 derivatives on the cellular growths of microbial pathogens and mammalian cells. The most biorelevant compounds are indicated in the scatter dot plot.

Table S1

Clinical isolate	**Antibiotic-resistance profile**
MRSA 1[§]	GMN^S, LVX^R, **OX^R**, **P^R**, TE^S, TEC^S, SXT^S, VA^S
MRSA 2[§]	GMN^S, LVX^R, **OX^R**, **P^R**, TE^S, TEC^S, SXT^S, VA^S
MRSA 3[§]	**CM^R, E^R,** GMN^S, **LVX^R**, **OX^R**, **P^R**, TEC^S, TE^S, SXT^S, VA^S
MRSA 4[§]	CM^S, E^S, GMN^S, **LVX^R**, **OX^R**, **P^R**, TE^S, TEC^S, SXT^S, VA^S
MRSA 5[§]	CM^S, E^S, GMN^S, **LVX^R**, **OX^R**, **P^R**, TE^S, TEC^S, SXT^S, VA^S
MSSA 1	CM^S, E^S, LVX^S, OX^S, **P^R**, TE^S, SXT^S
MSSA 2	**CM^R**, **E^R**, LVX^S, OX^S, P^S, TE^S, SXT^S
MSSA 3	CM^S, E^S, LVX^S, OX^S, **P^R**, TE^S, SXT^S
MSSA 4	**CM^R**, **E^R**, LVX^S, OX^S, **P^S**, TE^S, SXT^S
MSSA 5	CM^S, E^S, LVX^S, OX^S, **P^R**, TE^S, SXT^S

CM = Clindamicyn; E = Erythromycin; GMN = Gentamicin; P = Penicillin; LVX = Levofloxacin; OX = Oxacillin; TE = Tetracycline; TEC = Teicoplanin; SXT = Trimethoprim/Sulfamethoxazole; VA = Vancomycin
R = Resistant; S = Susceptible; I = Intermediate, as defined following the EUCAST guidelines
[§]*Staphylococcus* species resistant to oxacillin were declared, by convention, methicillin-resistant.
